# *N*,*N*-dimethylformamide tailors solvent effect to boost Zn anode reversibility in aqueous electrolyte

**DOI:** 10.1093/nsr/nwac051

**Published:** 2022-03-16

**Authors:** Yilin Ma, Qiu Zhang, Luojia Liu, Yixin Li, Haixia Li, Zhenhua Yan, Jun Chen

**Affiliations:** Key Laboratory of Advanced Energy Materials Chemistry (Ministry of Education), Renewable Energy Conversion and Storage Center (RECAST), College of Chemistry, Nankai University, Tianjin 300071, China; Key Laboratory of Advanced Energy Materials Chemistry (Ministry of Education), Renewable Energy Conversion and Storage Center (RECAST), College of Chemistry, Nankai University, Tianjin 300071, China; Key Laboratory of Advanced Energy Materials Chemistry (Ministry of Education), Renewable Energy Conversion and Storage Center (RECAST), College of Chemistry, Nankai University, Tianjin 300071, China; Key Laboratory of Advanced Energy Materials Chemistry (Ministry of Education), Renewable Energy Conversion and Storage Center (RECAST), College of Chemistry, Nankai University, Tianjin 300071, China; Key Laboratory of Advanced Energy Materials Chemistry (Ministry of Education), Renewable Energy Conversion and Storage Center (RECAST), College of Chemistry, Nankai University, Tianjin 300071, China; Key Laboratory of Advanced Energy Materials Chemistry (Ministry of Education), Renewable Energy Conversion and Storage Center (RECAST), College of Chemistry, Nankai University, Tianjin 300071, China; Key Laboratory of Advanced Energy Materials Chemistry (Ministry of Education), Renewable Energy Conversion and Storage Center (RECAST), College of Chemistry, Nankai University, Tianjin 300071, China

**Keywords:** aqueous electrolyte, Zn reversibility, hydrogen bond, solvent effect, rechargeable Zn battery

## Abstract

Rechargeable aqueous Zn batteries are considered as promising energy-storage devices because of their high capacity, environmental friendliness and low cost. However, the hydrogen evolution reaction and growth of dendritic Zn in common aqueous electrolytes severely restrict the application of Zn batteries. Here, we develop a simple strategy to suppress side reactions and boost the reversibility of the Zn electrode. By introducing 30% (volume fractions) *N*,*N*-dimethylformamide (DMF) to the 2 M Zn(CF_3_SO_3_)_2_–H_2_O electrolyte (ZHD30), the preferential hydrogen-bonding effect between DMF and H_2_O effectively reduces the water activity and hinders deprotonation of the electrolyte. The ZHD30 electrolyte improves the Zn plating/stripping coulombic efficiency from ∼95.3% to ∼99.4% and enhances the cycles from 65 to 300. The Zn–polyaniline full battery employing the ZHD30 electrolyte can operate over a wide temperature range from –40°C to +25°C and deliver capacities of 161.6, 127.4 and 65.8 mAh g^–1^ at 25, –20 and –40°C, respectively. This work provides insights into the role of tuning solvent effects in designing low-cost and effective aqueous electrolytes.

## Teaser

Strong hydrogen bonds effect between N,N-dimethylformamide and H_2_O reduces the water activity and enhances the performance of low-temperature aqueous Zn batteries.

## INTRODUCTION

To meet the application requirements of rapidly developing consumer electronics devices, electric automobiles and large-scale energy storage, it is urgent to develop new types of batteries that afford high capacity and are environmentally friendly and cost-effective [1,2]. Among all the available energy-storage devices, rechargeable aqueous Zn-ion batteries (RZBs) with high theoretical capacity (820 mAh g^–1^) of Zn anode, high safety and low cost have been regarded as promising candidates for energy storage [3–8]. However, Zn anodes suffer from intrinsic irreversibility issues [9–13]. In common slightly acidic electrolytes, the thermodynamic potential of the hydrogen evolution reaction (HER) (HER, 0 V vs. standard hydrogen electrode, SHE) significantly favors Zn deposition (–0.76 V vs. SHE). Consequently, the HER inevitably leads to consumption of the Zn anodes and swelling of the batteries, which results from fluctuations in the pH of the local environment and gas evolution [14,15]. Therefore, the construction of high-performance RZBs fundamentally requires inhibition of the HER.

Until now, strategies to suppress the HER have advanced, such as surface coating of the Zn anode to modify the electrode–electrolyte interface [16–18] and the design of nanostructured Zn anodes [19,20]. However, after these modifications, the electrolyte itself retains strong reductive ability to produce H_2_, which limits its further application. Considering the crucial role of the electrolyte, the highly concentrated electrolyte was developed to optimize the solvation structure [21–23]. It effectively suppresses the water activity, but leads to the high cost of the batteries [24]. Additionally, limited by intrinsic properties of water and excessive viscosity because of the high concentration, the ion transportation and interphase severely deteriorate in the concentrated electrolyte with temperature reduction, which restricts aqueous Zn batteries in storing and converting the renewable natural resources in cold regions and hinders their large-scale applications [25]. Although Xie *et al.* reported a ‘molecular crowding’ electrolyte that stabilized the water molecules in aqueous Li-ion batteries, preventing the reduction of H_2_O at the Zn anode remains challenging [26]. Recently, a relatively simple and universal solution has been used in which organic additives that reform the electrolytic structure are introduced into the electrolyte [13,27–29]. Notably, introducing organics may cause two major solvent effects in the electrolyte. First, the introduced organics can interact with water molecules, which significantly modulates the hydrogen-bond (HB) structure of the aqueous electrolyte. The organics may also be inserted into the inner solvation sheath of the cation and occupy the position of H_2_O inside the solvation sphere, resulting in the readjustment of the Zn^2+^ solvation structure. However, the priority of the two solvent effects and the mechanism of suppression of the HER are still undefined. Consequently, a multi-perspective approach is required to facilitate greater exploration of the relationship between the HER of the electrolyte with the amount of the organic additive and solvent effects, respectively. *N*,*N*-dimethylformamide (DMF) features a high donor number, low cost, high solubility in water and as an additive has the ability to reform the electrolyte structure. Wang *et al.* reported a Zn battery employing a single solution of DMF and confirmed that DMF-based electrolytes have good compatibility with Zn anodes [30]. Notably, pure DMF is a potential safety hazard because of its high flammability; it can be introduced into water to formulate a much safer electrolyte. DMF owns lone-pair electrons on the O atom, enabling it to form strong interaction with H_2_O as the HB acceptor. With strong electron-donating ability, DMF can be used to regulate the structure of the electrolyte and reduce water activity [31]. However, the relevant mechanism through which these processes are achieved has not been revealed [32].

**Figure 1.**
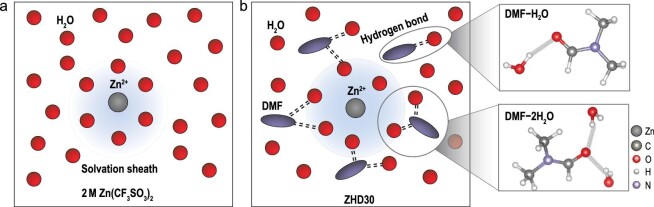
Scheme of inner electrolyte structure and solvent effect in (a) 2 M Zn(CF_3_SO_3_)_2_ and (b) ZHD30 electrolyte.

Herein, by introducing different amounts of DMF into the 2 M Zn(CF_3_SO_3_)_2_–H_2_O electrolyte (ZHD), the priorities and impacts of two solvent effects on the HER, namely the re-establishment of HBs and modulation of the inner solvation sheath by DMF, are explored. We discovered that DMF (volume fraction ≤30%) can preferentially regulate the HB networks in the electrolyte, which effectively inhibits the deprotonation of water. With the increase in DMF added (volume fraction >30%), the DMF begins to insert into the Zn^2+^ solvation sheath except for forming HBs with water and reduces the electrochemical stability of the electrolyte. Thus, the ZHD30 (with 30% DMF) electrolyte with a critical concentration (DMF volume ratio = 30%) can be used to achieve optimal Zn reversibility. In the ZHD30 electrolyte, DMF reconstructs the HB network between the O in DMF and the H in water, considering that it does not enter the first coordination shell of Zn^2+^, and therefore facilitates maximum thermodynamic reduction in the water activity (Fig. 1). Inhibition of the HER can enhance the Coulombic efficiency (CE) of Zn plating/stripping to ∼99.4% (300 cycles) and extend the cycle life of the Zn‖Zn symmetric cells to 2000 h. Additionally, the re-established HB networks suppress the ice nucleation process at low temperatures and improve the environmental adaptability of the electrolyte. The Zn–polyaniline (PANI) full battery based on this highly reversible electrolyte is demonstrated to operate over a wide temperature range from –40°C to +25°C. This study provides insight into the suppression of interfacial parasitic reactions via the solvent effect and offers a novel strategy for designing low-cost and effective aqueous electrolytes.

## RESULT AND DISCUSSION

**Figure 2.**
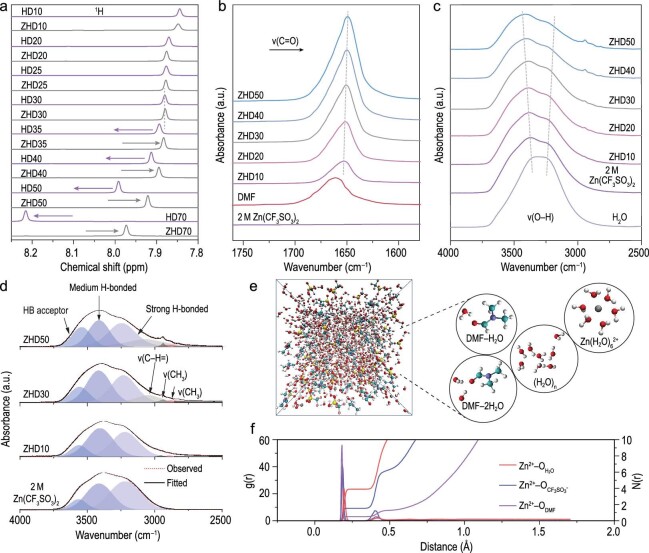
(a) The ^1^H chemical shifts of H_2_O–DMF and Zn(CF_3_SO_3_)_2_–H_2_O–DMF at different volume fractions. FT–IR of Zn(CF_3_SO_3_)_2_–D_2_O–DMF at different volume fractions. (b) C=O band of DMF. FT–IR of Zn(CF_3_SO_3_)_2_–H_2_O–DMF at different volume fractions. (c) O–H stretching band of water. (d) The fitted FTIR spectra of O–H stretching band of water. (e) A snapshot of ZHD30 electrolyte in MD simulation. (f) The RDF and coordination number among Zn^2+^, H_2_O, anions and DMF.

Fourier-transform infrared (FTIR), nuclear magnetic resonance (NMR) and molecular dynamics (MD) simulations were used to explore the interactions between DMF, water and Zn^2+^. Appreciable heat was generated in the aqueous solution after DMF was added to pure water (Supplementary Fig. 1), but no additional peaks appeared in the FTIR spectrum of H_2_O–DMF (Supplementary Fig. 2). This heat generation suggests that HBs were formed between DMF and the water molecules. To further study the priority of different solvent effects, the ^1^H chemical shift of the aldehyde hydrogen on DMF was utilized as the sensitive indicator. In Fig. 2a, as the content of DMF increased, the ^1^H signal in the H_2_O–DMF mixed solution (HD) started shifting to the low field. This shift resulted from hydrogen bonding between the O atom in DMF and the H atom in the water molecules (C=O···H–O), which deshielded the nucleus of the aldehydic hydrogen [21]. When the Zn(CF_3_SO_3_)_2_ salt (2 M) was added to the HD with a low amount of DMF, the downshift in the ^1^H signal in the ZHD electrolyte was consistent with the downshift in the HD, which confirmed that that there is only the HB effect of DMF. When the volumetric ratio of DMF increased (>30%), the ^1^H signal in ZHD began to shift to a high field. This shift resulted from the involvement of DMF in the Zn^2+^ solvation structure, which increased the electron density around the nucleus of the aldehydic hydrogen and further resulted in an enhanced shielding effect [33].

Figure 2b shows the regional FTIR spectra of ZHD electrolyte with different DMF volume fractions (full FTIR spectra displayed in Supplementary Fig. 3). It can be found that the ZHD electrolyte shows a unique stretching mode of the carbonyl (C=O) group in 1660 cm^–1^. As the DMF content increased (<30%), the spectral band of the ZHD electrolyte shifted to a lower wave number (red-shift), which could be explained by the water trapped by DMF via HB interactions. With a further increase in the DMF fraction, the DMF molecules involved in the solvation sheath began to supersede the water molecules, which weakened the C=O bond, resulting in a further red-shift to 1647 cm^–1^. The O–H stretching vibration of H_2_O at 2500–4000 cm^–1^ is shown in Fig. 2c. Based on previous reports, the stretching vibration can be deconvoluted into three components at 3230.2, 3417.9 and 3552.7 cm^–1^, which represent the intermolecular interactions of H_2_O via strong H_2_O–H_2_O HBs, medium H_2_O–H_2_O HBs and the H_2_O bound to the HB acceptor, respectively [34,35]. As the content of DMF increased, the ratio of water in different H-bound states was calculated from the area of the fitted peaks. Quantification of the different hydrogen-bonded states indicated that water molecules combined with the HB acceptor dramatically increased, demonstrating that the DMF addition successfully manipulated the initial HB network in ZHD electrolytes (Fig. 2d).

Additionally, to reveal the internal Zn^2+^ coordination environment and HB structure inside, MD was used to simulate the ZHD electrolyte. Taking ZHD30 electrolyte as an example, there are four structures, including (H_2_O)_n_, Zn(H_2_O)_6_^2+^, DMF–H_2_O and DMF–2H_2_O (Fig. 2e). As shown in the radial distribution function (RDF), the corresponding coordination number of ZHD30 indicated that the solvation configuration of Zn^2+^ was dominated by the aqua ions Zn(H_2_O)_6_^2+^ (Fig. 2f). Moreover, a large amount of DMF combined with H_2_O to form HBs, while a small amount of DMF replaced the H_2_O in the Zn^2+^ solvation sheath (Supplementary Fig. 4). Based on the MD simulations and the NMR spectra, introducing DMF into the aqueous electrolyte generated a HB effect with water, which took precedence over the coordination effect with Zn^2+^.

**Figure 3.**
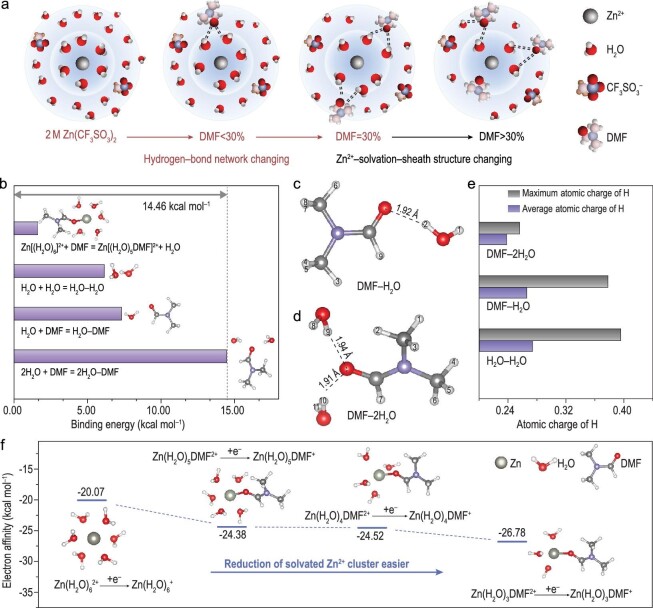
(a) Schematic of changes in Zn^2+^ solvent sheath and local hydrogen-bond network with DMF addition. (b) Binding energies among DMF, H_2_O and Zn^2+^. (c) and (d) Major configurations when DMF binds with H_2_O. (e) The average and maximum atomic charge of H atoms in different configurations. (f) Electron affinity calculations of different Zn^2+^ solvation sheath.

Figure 3a illustrates the evolution of the solvent effect in the 2 M Zn(CF_3_SO_3_)_2_ electrolyte after DMF was added. In the 2 M Zn(CF_3_SO_3_)_2_ electrolyte without DMF, aqua ions Zn(H_2_O)_6_^2+^ dominated the solvation structure. After it was introduced, DMF first combined with H_2_O to form HBs. As the volume of DMF increased to >30%, DMF was progressively inserted into the outer and inner solvation sheaths of Zn^2+^, which significantly affected the coordination balance of Zn^2+^. Density functional theory was used to calculate the binding energies of DMF, H_2_O and Zn^2+^ (Fig. 3b). Owing to the lone pair of electrons on the O atom of DMF, the binding energy between DMF and H_2_O is 14.46 kcal mol^–1^ (DMF–2H_2_O) and 7.31 kcal mol^–1^ (DMF–H_2_O), which is higher than the energy required for DMF to replace H_2_O in Zn(H_2_O)_6_^2+^ (1.63 kcal mol^–1^). The difference in the binding energy determines the priorities of the two solvent effects, considering that DMF preferentially forms HBs with water molecules in the ZHD electrolyte. To unveil the mechanism of water stabilization in the DMF-based electrolyte, the basic properties of H_2_O in different solvation configurations were calculated and compared. Figure 3c and d shows two major configurations in the ZHD electrolyte, namely DMF–H_2_O and DMF–2H_2_O. The atomic charge on the H atoms revealed the effect of water–DMF interactions on deprotonation (Supplementary Fig. 5). The average atomic charge and maximum atomic charge of the H atoms in DMF–2H_2_O (0.32, 0.33) and DMF–H_2_O (0.33, 0.39) were lower than those of the H atoms in H_2_O–H_2_O, which proves that the HBs generated by DMF can reduce the tendency of H_2_O to deprotonate and hinder the HER of the electrolyte (Fig. 3e). The difference in the electron transfer for H_2_O–H_2_O, DMF–H_2_O and DMF–2H_2_O changes the O–H bond order. As a result, the O–H bond orders increase from (0.89, 0.89, 0.92, 0.81) in the H_2_O–H_2_O state to (0.93, 0.87) in the DMF–H_2_O state and (0.92, 0.87, 0.94, 0.84) in the DMF–2H_2_O state, indicating that the HB interaction in DMF–H_2_O will lead to the formation of more stable O–H bonds (Supplementary Fig. 6). The influence of the two solvent effects on the electrochemical stability of the electrolyte was further revealed. As shown in Fig. 3f, we compared the electron affinity among the initial Zn^2+^ solvation sheath (Zn(H_2_O)_6_^2+^) and the altered solvation sheath in which different addition of DMF participates. When compared with Zn(H_2_O)_6_^2+^, the electron affinity of Zn(H_2_O)_5_DMF^2+^, Zn(H_2_O)_4_DMF^2+^ and Zn(H_2_O)_3_DMF^2+^ decreased, indicating that the Zn^2+^ solvation sheath was unstable after DMF was added and the overall stability of the ZHD electrolyte (where the volumetric ratio of DMF was >30%) was reduced. The combination of spectra and theoretical calculations suggests that DMF preferentially formed HBs with H_2_O in the electrolyte, which suppressed the water activity and inhibited the deprotonation of water. Notably, the entry of DMF into the Zn^2+^ solvation structure was not conducive to stability. Thus, the amount of DMF introduced should be controlled at a critical concentration that is optimal for thermodynamic suppression of the HER. This concentration should facilitate the formation of extensive HBs of DMF with water molecules without involving DMF in the Zn^2+^ solvation sheath. A DMF concentration of 30% (ZHD30) is the critical point at which the solvent effect induced by DMF from the single HB effect leads to the coexistence of both the coordination effect and the HB effect. Therefore, ZHD30 was chosen as the optimal electrolyte to further explore its electrochemical performance.

**Figure 4.**
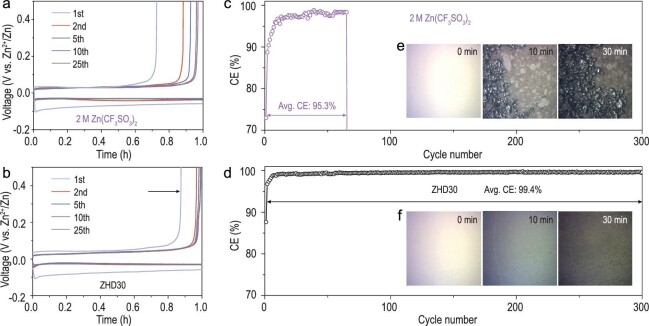
Voltage profiles and CE of Zn plating/stripping in Zn‖Cu batteries with (a) and (c) 2 M Zn(CF_3_SO_3_)_2_ electrolyte and (b) and (d) ZHD30 electrolyte with a current density of 0.2 mA cm^−2^ and an areal capacity of 0.2 mAh cm^−2^. Insets of (e) and (f) are the images captured by an AFM optical camera during Zn plating processes.

Considering that the organic solvents owning different electron-donating ability can reconstruct the electrolyte structure and eliminate the HER side reactions, various species of organic solvents, such as dimethylsulfoxide (DMSO), dimethoxyethane (DME) and dimethyl carbonate (DMC), were chosen for the comparison of the reduction stability of organic solvent–water complexes. The partial density of states (PDOS) of H_2_O binding with different organic solvents were further calculated. The addition of DMF achieved better improvement in stabilizing water molecules than other organic solvents, as indicated by the higher lowest unoccupied molecular orbital energy level of H_2_O increasing obviously in the DMF–H_2_O state (Supplementary Fig. 7). To demonstrate the availability of ZHD30 electrolyte in RZBs, the ionic conductivity, viscosity and electrochemical stability window of the electrolyte and the cyclic voltammetry (CV) responses of the Zn‖Ti asymmetric cells were tested (Supplementary Figs 8–10). Furthermore, the impact of adding DMF on the reversibility of the Zn electrode was investigated by using Zn‖Cu asymmetric cells under galvanostatic conditions. CE in the first cycle can avoid the influence of competitive side reactions during the cycling, which is an important parameter for investigating the intrinsic chemical reversibility of Zn. In Fig. 4a, the first-cycle CE based on 2 M Zn(CF_3_SO_3_)_2_ was only 73.0%. Due to the high water activity in this conventionally dilute solution, the HER occurred during the Zn plating/stripping process, which decreased the CE in the first cycle. In addition, the unstable stripping signal of 2 M Zn(CF_3_SO_3_)_2_ was observed in <65 cycles with an average CE value of 95.3% as a result of the occurrence of competitive side reactions such as corrosion and dendrite formation (Fig. 4c). By contrast, with ZHD30, the CE in the first cycle increased significantly to 87.7%, which strongly improved the intrinsic ability of the electrolyte to prevent reduction (Fig. 4b). Furthermore, benefiting from the regulation of the HB networks and reduced activity of water molecules in the ZHD30 electrolyte, the CE of the Zn anode increased rapidly in the first 10 cycles and remained stable after 300 cycles, which finally obtained a high average value of 99.4% (Fig. 4d). Notably, the re-established and unstable Zn solvation structure in ZHD50 led to a lower average CE for the Zn anode when compared to the CE in ZHD30, which was only ∼92.7% (Supplementary Fig. 11).

To intuitively explore the inhibition of DMF additive on HER, corrosion and passivation in the electrolyte, *in situ* atomic force microscope (AFM) equipped with an optical camera was used to obtain the optical image of the electrode surface during Zn plating. As shown in Fig. 4e, in 2 M Zn(CF_3_SO_3_)_2_, with the formation of hydrogen during the Zn plating process, the gold layer on the electrode easily peeled off the electrode and the HER caused notable protuberances in the system. Simultaneously, as the density of gas bubbles increased with the generation of hydrogen, the gold layer became disconnected from the electrode, which was not conducive to Zn deposition. In contrast, after DMF was introduced, due to the reconstruction of HB networks, the deprotonation tendency of water was alleviated and the evolution of hydrogen was weakened (Fig. 4f).

**Figure 5.**
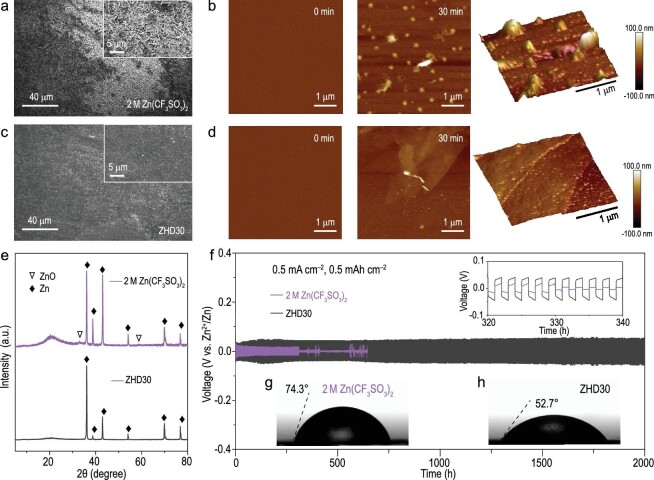
SEM images and magnified SEM images of the deposited Zn metal in (a) 2 M Zn(CF_3_SO_3_)_2_ electrolyte and (c) ZHD30 electrolyte after the 50th plating. AFM images of Zn nucleation and growth in (b) 2 M Zn(CF_3_SO_3_)_2_ electrolyte and (d) ZHD30 electrolyte during the initial Zn electrodeposition. (e) XRD patterns of Zn anodes after plating/stripping cycles in Zn(CF_3_SO_3_)_2_ and ZHD30 electrolytes. (f) Galvanostatic Zn plating/stripping curves in Zn‖Zn symmetric cells at a current density of 0.5 mA cm^−2^ and an areal capacity of 0.5 mAh cm^−2^. Contact-angle measurement on Zn in (g) 2 M Zn(CF_3_SO_3_)_2_ electrolyte and (h) ZHD30 electrolyte.

To further evaluate the H_2_O reduction behavior in the Zn plating process, gas chromatography with a designed cell was applied to monitor the gas evolution in the electrolytes with and without DMF. Compared with the 2 M Zn(CF_3_SO_3_)_2_ electrolyte, the ZHD30 electrolyte induced notable suppression of the HER (Supplementary Fig. 12). Based on the scanning electron microscope (SEM) image, it can be seen that the nano-flake layer covered the Zn electrode after cycling in the 2 M Zn(CF_3_SO_3_)_2_ electrolyte (Fig. 5a). X-ray powder diffraction (XRD) characterization (Fig. 5e) further confirmed that this layer consisted of ZnO. This significant corrosion and passivation had a significant impact on the CE of Zn plating/stripping and ultimately damaged the Zn electrode. On the contrary, under the same test conditions, the surface of the Zn anode still exhibited a flat and dendrite-free morphology after 50 cycles in the ZHD30 electrolyte (Fig. 5c). The uniform Zn deposition promoted the reversibility of Zn. This finding confirmed that the Zn plating/stripping behavior in the aqueous electrolyte was improved after DMF was added. AFM was also used to visualize the Zn deposition. Influenced by the tip effect, Zn^2+^ ions in the 2 M Zn(CF_3_SO_3_)_2_ electrolyte were preferentially deposited on the tiny protuberances at the initial stage of formation of the Zn nuclei. After galvanostatic plating at 50 μA cm^–2^ for 30 mins, the Zn^2+^ ions aggregated at the nucleation sites and finally evolved into a 3D stacked dendrite, which damaged the surface and degraded Zn utilization (Fig. 5b). By contrast, the prominently changing Zn plating/stripping behavior was captured in the ZHD30 electrolyte. Unlike the vertically grown Zn dendrites, Zn ions in the ZHD30 electrolyte exhibited homogeneous nucleation and diffusion, which facilitated even Zn distribution and dendrite-free Zn deposition (Fig. 5d). The influence of adding DMF on the cycling stability of the Zn anode was further evaluated in a Zn‖Zn symmetric battery. Figure 5f shows the potential–time curve of the Zn‖Zn battery at a current density of 0.5 mA cm^–2^ and a capacity of 0.5 mAh cm^–2^. The Zn‖Zn battery with the Zn(CF_3_SO_3_)_2_ electrolyte (2 M) could only operate stably for 312 h, with an overpotential of ∼20.0 mV. Thereafter, due to the interference of HER and the growth of Zn dendrites, the polarization voltage suddenly and irreversibly increased, which eventually short-circuited the battery. In contrast, the ZHD30-based Zn‖Zn battery exhibited a 5-fold longer cycle life (≤2000 h at 0.5 mA cm^–2^). Although the introduction of DMF inevitably reduced the ionic conductivity and viscosity of the electrolyte and increased the polarization of the battery by ∼26 mV, it effectively inhibited side reactions and resulted in good compatibility between ZHD30 and the Zn metal anode. A comparison of the present system with recently reported strategies for boosting Zn reversibility is summarized in Table S5, which shows that in terms of the CE and cycle life, the ZHD30 electrolyte exhibited competitive electrochemical performance. The rate performance of the Zn‖Zn symmetric cells with the ZHD30 electrolyte was also tested. Because of the homogeneous nucleation of Zn, the cells maintained a high areal capacity of 5 mAh cm^–2^ (Supplementary Figs 13–15). The universality of introducing DMF for improving the utilization of Zn was further investigated using 2 M ZnSO_4_ aqueous electrolyte. The Zn‖Zn symmetric cell without DMF showed a sudden overpotential within 58 h. On the contrary, the cell employing ZnSO_4_ (2 M) electrolyte with DMF (30%) cycled steadily with a lower overpotential over 100 h, confirming the effectiveness of adding DMF in boosting the Zn reversibility (Supplementary Fig. 16).

The wettability of the Zn surface directly affected the energy barrier of the initial nucleation and growth of Zn. Therefore, the contact-angle technique was used to evaluate the influence of adding DMF on the wettability of the electrode. Owing to its hydrophobicity, Zn metal had a contact angle as high as 74.3° in the 2 M Zn(CF_3_SO_3_)_2_ electrolyte (Fig. 5g). However, after the introduction of DMF (30%), the contact angle was reduced to 52.7°, which indicates that DMF exhibits high wettability for Zn metal (Fig. 5h). The low contact angle suggests that DMF may be preferentially adsorbed on the electrode surface, thus suppressing corrosion and passivation, which effectively improved the CE and cycling stability of the Zn anode. Notably, although flammable DMF was introduced, the ZHD30 electrolyte still contained sufficient H_2_O, retaining the ability to resist burning. Owing to the addition of highly flammable pure DMF, the safety test was carried out to confirm the safe concentration of DMF. When the volumetric ratio of DMF : H_2_O was ≤9 : 1, the hybrid electrolyte was safe and nonflammable (Supplementary Fig. 17). DSC was used to investigate the thermodynamic behavior of the as-prepared ZHD30 electrolyte (Supplementary Fig. 18). Although increasing the DMF content led to higher viscosities, the reconstructed HB networks guaranteed a decreasing freezing point of the aqueous electrolyte to –35°C, indicating that the electrolyte is adaptable to harsh environments [36,37].

Zn‖Zn symmetric batteries were used to evaluate the compatibility of Zn with the ZHD30 electrolyte under subzero conditions. The Zn‖Zn battery with the ZHD30 electrolyte showed stable and continuous operation at –40°C (Supplementary Fig. 19). In comparison, there was a notable short circuit in the Zn‖Zn battery with the 2 M Zn(CF_3_SO_3_)_2_ electrolyte at –30°C due to dendrite growth (Supplementary Fig. 20). SEM and XRD were used to characterize the Zn electrode surface in the ZHD30 electrolyte after the 10th and 50th plating at –40°C (Supplementary Figs 21 and 22). A compact morphology and no by-products were observed, which indicated that there was high Zn reversibility in the ZHD30 electrolyte at low temperatures.

**Figure 6.**
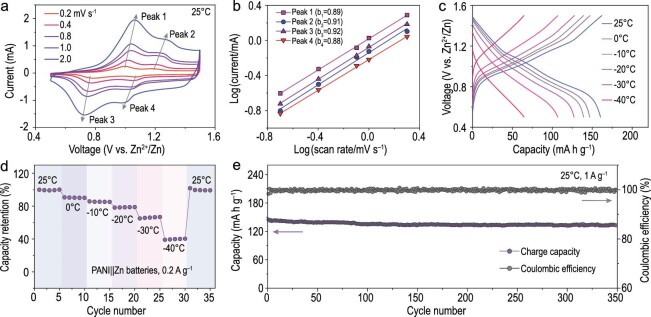
(a) CV curves of the Zn|ZHD30|PANI battery. (b) The corresponding plots of log(peak current) vs. log(scan rate). (c) Discharge–charge curves of Zn|ZHD30|PANI battery at the temperature range from –40°C to +20°C. (d) The electrochemical performance of the Zn|ZHD30|PANI battery in varying temperature. (e) Cycling performance of the Zn|ZHD30|PANI battery.

To meet the demands of large-scale practical applications, organic materials for wide-temperature-range batteries have received attention [38] owing to their special charge-storage mechanisms mainly located at surface groups, which is different from the sluggish insertion kinetics of conventional inorganic materials [39,40]. Therefore, polyaniline (PANI) was chosen to construct a full battery. The related material characterizations are shown in Supplementary Figs 23 and 24. Figure 6a shows the CV curves of the Zn|ZHD30|PANI battery at different scan rates from 0.2 to 2.0 mV s^–1^. The equation log(*i*) = log(*a*) + *b* × log(*v*) was used to fit the *b*-value, where a *b*-value close to 1 indicates that pseudo-capacitive behavior is dominant during the charging–discharging, while a *b*-value close to 0.5 indicates that the reaction was controlled by ion diffusion [4]. Figure 6b shows that the *b*-values of the peaks are close to 1, which indicates that the electrochemical reaction in the Zn|ZHD30|PANI battery allowed predominantly pseudocapacitance behavior. This organic polymer electrode prevented the desolvation of solvated ions, which demonstrates its potential for application at low temperatures [39]. The Zn|ZHD30|PANI battery was subjected to charge–discharge cycles from −40°C to 25°C (Fig. 6c and d). When the current density was 200 mA g^–1^, the battery exhibited a reversible capacity of 161.6 mAh g^–1^ at room temperature. Under subzero conditions, the battery retained high capacities of 147.7, 139.6, 127.4, 105.9 and 65.8 mAh g^–1^ at 0°C, –10°C, –20°C, –30°C and –40°C, respectively. Based on ZHD30 electrolyte, the battery still exhibited the high capacity retention of 65.5% at –30°C and 40.7% at –40°C, which demonstrated that the battery possessed excellent environmental adaptability. The long-term cycling stability of the Zn|ZHD30|PANI battery was subsequently evaluated at room temperature (Fig. 6e). The capacity of the Zn‖PANI cells in the ZHD30 electrolyte remained stable at 133.1 mAh g^−1^ at 1 A g^–1^ for 350 cycles, with a high capacity retention rate of 91.6%. The results demonstrate the superior performance, promising application and feasibility of ZHD30-based Zn batteries.

## CONCLUSIONS

In summary, we demonstrate that the DMF addition preferentially formed HBs with H_2_O in a hybrid electrolyte that successfully reduced the water activity of the electrolyte and inhibited the deprotonation of water. However, the insertion of excess DMF into the Zn^2+^ solvation structure deteriorated the stability of the electrolyte. Considering the relationship between the amount of DMF and thermodynamics suppression of HER, the optimal ZHD30 electrolyte was developed. The addition of  DMF (30%) ensured a high CE of ∼99.4% up to 300 cycles during Zn plating/stripping, which enabled a cycle life of 2000 h for Zn‖Zn symmetric cells. Furthermore, the addition of DMF (30%) induced high compatibility of the electrolyte with the Zn anodes under subzero conditions. The Zn|ZHD30|PANI battery operated over a wide temperature range of –40°C to +25°C, with discharge capacities of 147.7, 139.6, 127.4, 105.9 and 65.8 mAh g^–1^ at 0°C, –10°C, –20°C, –30°C and –40°C, respectively, stable cycling performance and a high capacity retention of 91.6% after 350 cycles.

## MATERIALS AND METHODS

### Sample preparation

Zn foil (>99.99%) with a thickness of 30 μm was purchased from Ailian, Tianjin. Zn(CF_3_SO_3_)_2_ (>98%), NaCF_3_SO_3_(>98%) and DMF (>99.5%) were purchased from Aladdin and used without further purification. Mixtures of DMF and Zn(CF_3_SO_3_)_2_ (2 M) aqueous solutions were prepared from DMF–H_2_O with volumetric ratios of 0 : 10, 1 : 9, 2 : 8, 2.5 : 7.5, 3 : 7, 3.5 : 6.5, 4 : 6, 5 : 5, 7 : 3, 9 : 1 and 9.5 : 0.5; the samples are denoted as 2 M Zn(CF_3_SO_3_)_2_, ZHD10, ZHD20, ZHD25, ZHD30, ZHD35, ZHD40, ZHD50, ZHD70, ZHD90 and ZHD95, respectively. An ammonium persulfate oxidizing aniline monomer in aqueous hydrochloric acid was used to synthesize PANI, which was similar to the previously reported method [41].

### Electrochemical tests

The symmetric Zn‖Zn/asymmetric Zn‖Cu/Zn‖Ti batteries and PANI‖Zn batteries using CR2032 coin-type cells were composed of 30 μL ZHD electrolyte, Zn/Cu/Ti electrode (Φ12 mm) and filter paper as a separator. A mixture of PANI (60 wt%), Ketjen black (35 wt%) and polytetrafluoroethylene (5 wt%) was loaded onto a Ti mesh to prepare the PANI electrode. The discharge–charge profiles were obtained using LAND CT2000A. A CHI760E instrument was used to obtain linear sweep voltammetry and CV curves. The low-temperature performance was evaluated using a Meiling refrigerator DW–HW50 and a Suoyate WuXi chamber.

### Theoretical calculations

MD simulations for the electrolyte structures including Zn^2+^ coordination shell and water–DMF complexes were conducted by using the GROMACS package [42] with AMBER03 force field [43]. The MD parameter for Zn^2+^ was in the built-in force-field parameters. Water molecules were simulated in the TIP4P mode [44]. The MD parameters for CF_3_SO_3_^−^ and DMF were generated using ACPYPE [45] and the corresponding atom charges were based on the restrained electrostatic potential (RESP) charges [46]. The constant-pressure and temperature (NPT) runs were first performed at 298.15 K for 40 ns to ensure system equilibrium and then another NPT run of 10 ns was used for the analysis. The RDFs were calculated from the built-in module in the GROMACS package. The MD simulation snapshot was using visual molecular dynamics (VMD) [47]. The parameters of the simulated ZHD30 electrolyte are listed in Table S1. Quantum chemistry calculations were performed using the Gaussian 16 (Version A.03) program package. The structure optimization and frequency analysis were carried out at B3LYP/6–31G(d, p) level of theory. Among them, Zn atoms uniformly adopt the LANL2DZ pseudopotential basis set for accurate description of relativistic effect. Meanwhile, to simulate the solvation effect, single-point energy calculations were performed with consideration of implicit water solvent using the solvation model density (SMD) solvation model and the basis set was further enlarged to 6–311 + G(d, p). The O−H bond orders were acquired based on the Mayer bond order by the further calculation of Gaussian check files on Multiwfn [48,49]. PDOS were utilized for analysing the state of the water molecule in the organic solvent–water complexes, which was obtained by the further calculation of Gaussian check files on Multiwfn [49].

### Materials and characterization

See Supplementary Data section for details.

## Supplementary Material

nwac051_Supplemental_File
