# Supplementary material for: N,N-dimethylformamide tailors solvent effect to boost Zn anode reversibility in aqueous electrolyte
Source: Natl Sci Rev. 2022 Mar 16;9(10):nwac051. doi: 10.1093/nsr/nwac051 (PMC9671663; doi:10.1093/nsr/nwac051)
Supplement: nwac051_Supplemental_File [file nwac051_supplemental_file.docx]

**Supplemental Information**

***N,N*–dimethylformamide tailors solvent effects to boost Zn anode reversibility in aqueous electrolyte**

Yilin Ma, Qiu Zhang, Luojia Liu, Yixin Li, Haixia Li, Zhenhua Yan^*^, and Jun Chen^*^

Key Laboratory of Advanced Energy Materials Chemistry (Ministry of Education), Renewable Energy Conversion and Storage Center (RECAST), College of Chemistry, Nankai University, Tianjin 300071, China

*Correspondence: [yzh@nankai.edu.cn](mailto:yzh@nankai.edu.cn); chenabc@nankai.edu.cn

**Materials and Characterization**

Nuclear magnetic resonance (NMR) was characterized on Bruker ASCEND400. Fourier transform infrared spectroscopy (FTIR) was conducted on a Bruker Tensor II. The surface morphology of Zn anodes was characterized by scanning electron microscopy (SEM, IEOL JSM–7500F). The contact angle and surface tension were obtained by the contact angle system OCA 25 (DataPhysics). Galvanostatic Zn deposition (–0.04 mA cm^–2^) were carried out by atomic force microscope (AFM, Bruker Dimension Icon). The homemade in situ AFM cell contained a Zn metal electrode and a planar Au substrate. Zn electrode and Au electrode were used as the counter/reference and working electrodes, respectively. ScanAsyst mode and SNL-C probes (triangular silicon nitride, a force constant of 0.7 N m^–1^) were used to obtain height images. X–ray diffraction (XRD, Rigaku SmartLab instrument) was carried out to characterize the deposition. Differential Scanning Calorimetry (DSC) was obtained in METTLER TOLEDO DSC3. The cooling procedure was from +25 to –150 °C with a rate of 10 K min^–1^, keeping constant temperature at –150 °C for 2 minutes and heating rate of 5 K min^–1^ from –150 to +25 °C.


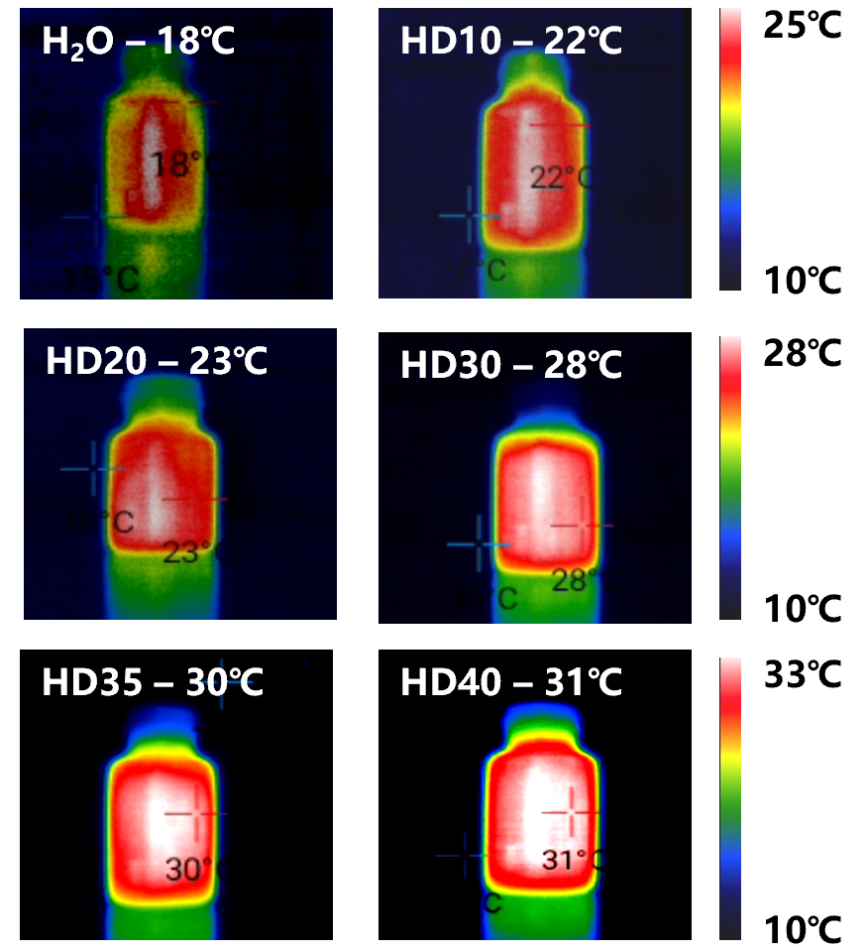


**Fig. S1.** Thermal imaging pictures when different volume fractions of DMF and water are mixed (different areas may have different temperatures).


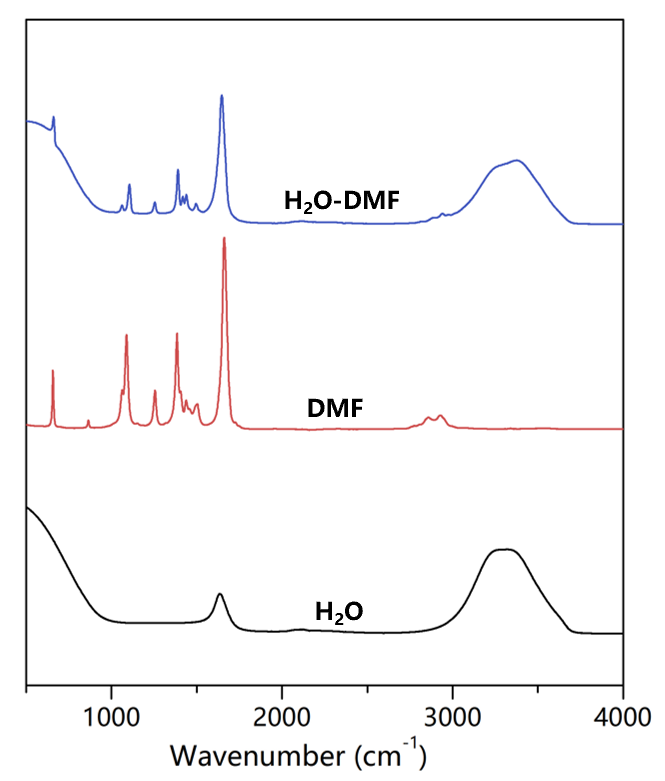


**Fig. S2.** FTIR of H_2_O, DMF and H_2_O-DMF mixed solutions.


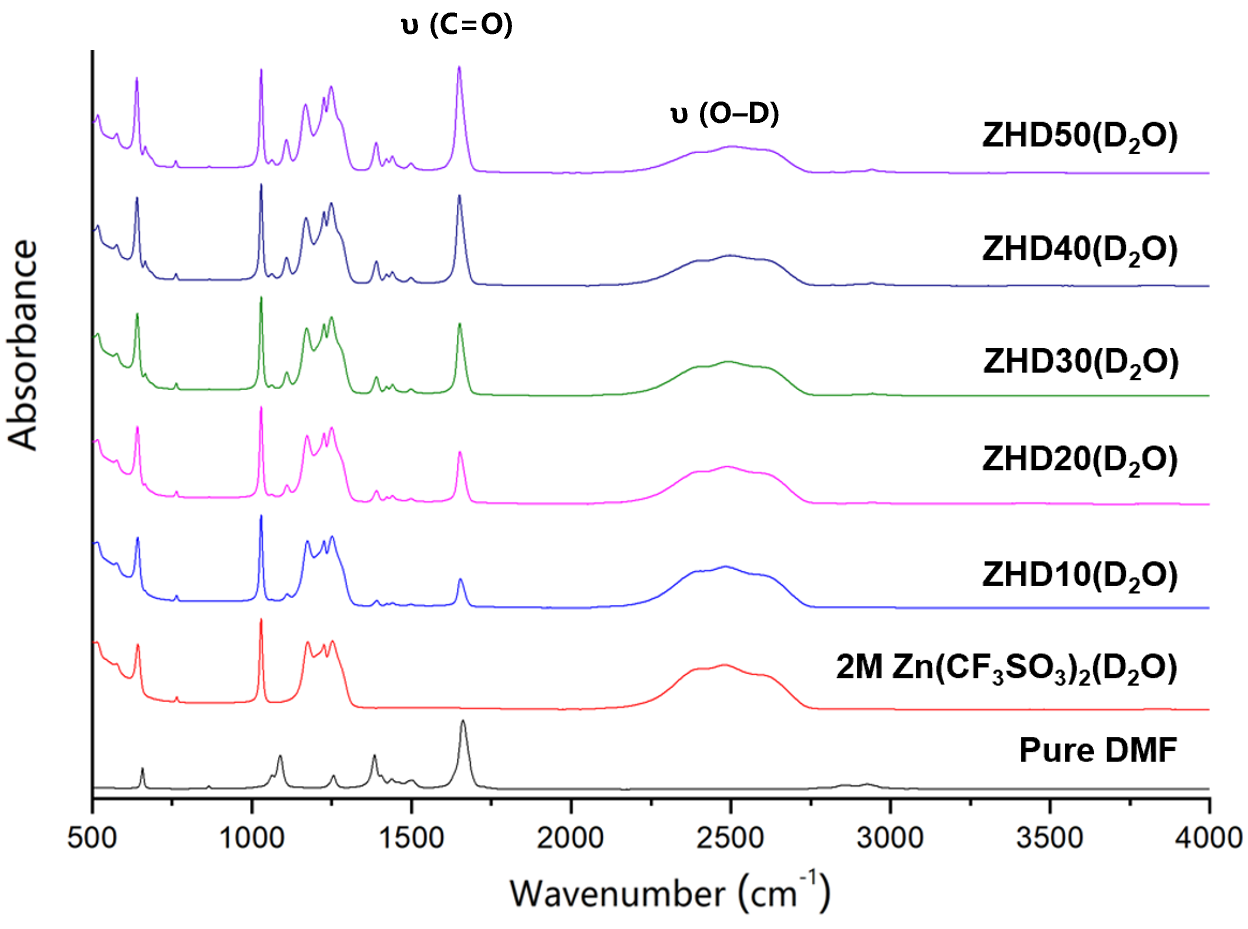


**Fig. S3.** Full FTIR spectra of DMF and different ZHD electrolyte.

The position of the δ(H-O-H) bending vibrations of water is close to that υ(C=O) stretching vibrations of DMF, which results in the misleading overlap. D_2_O has two more neutrons, similar geometrical configuration but different vibration frequency from H_2_O. Considering that D_2_O has no characteristic peak of δ(H-O-H) around 1630cm^-1^, using it instead of H_2_O for C=O stretching band analysis.


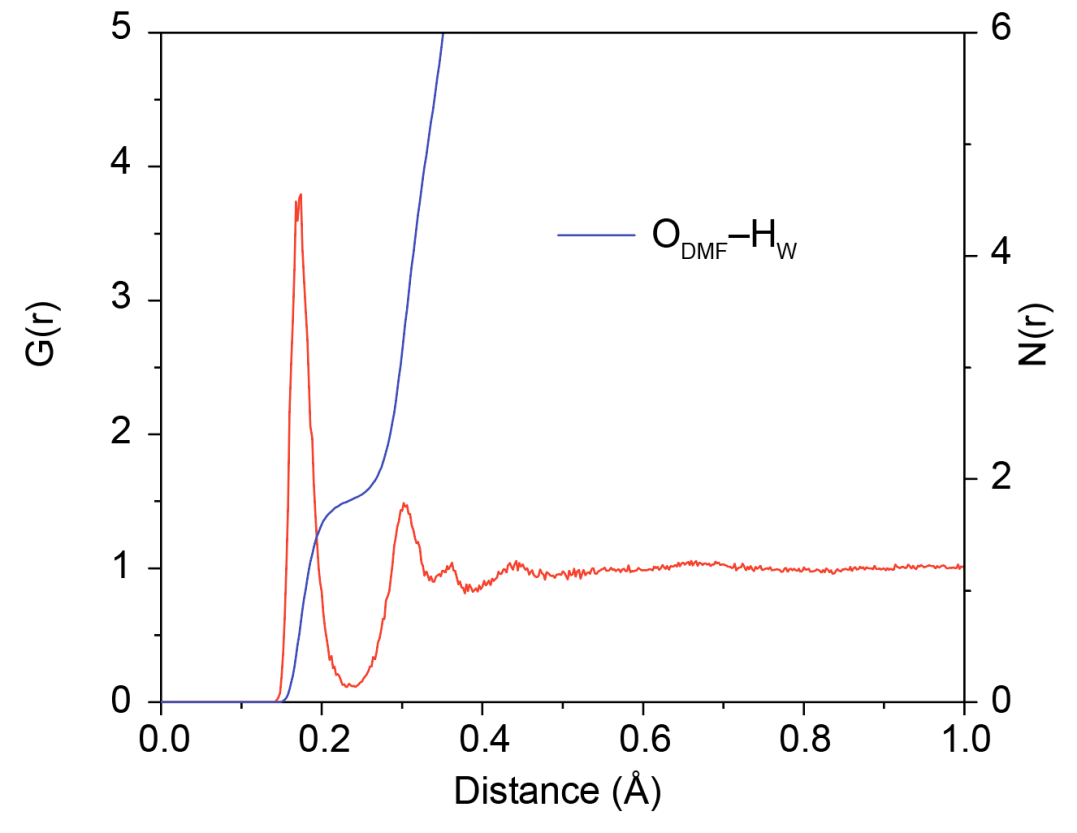


**Fig. S4.** The RDF and coordination number between the O atoms of DMF and the H atoms of H_2_O.


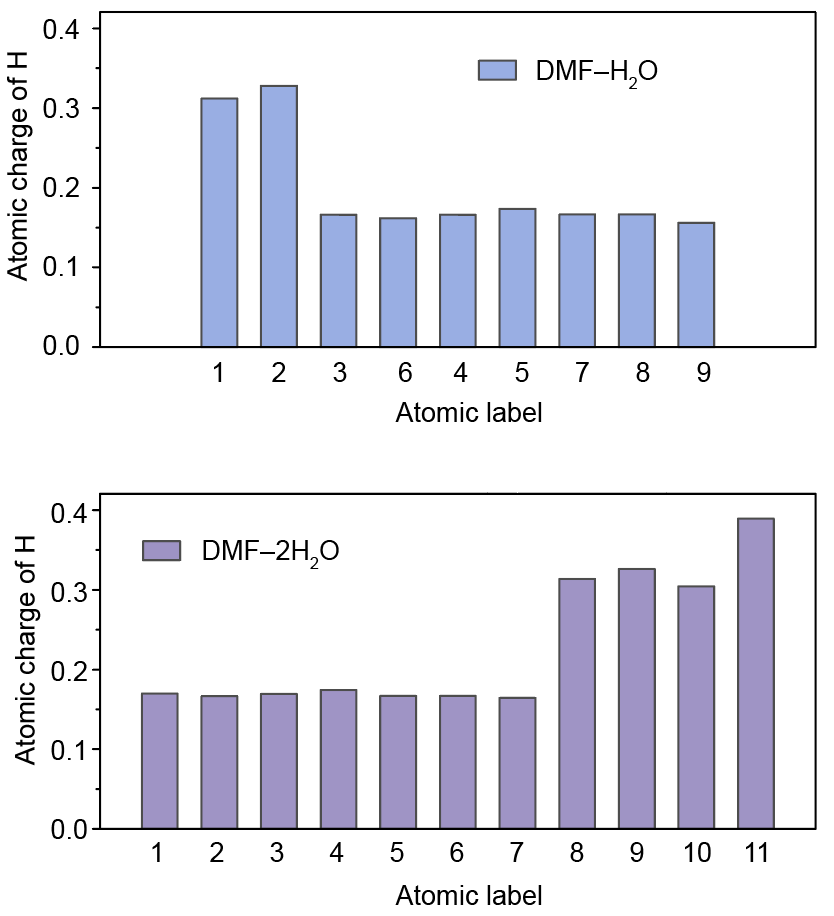


**Fig. S5.** The atomic charge of every H atom in DMF–H_2_O (up), and DMF–2H_2_O (down).


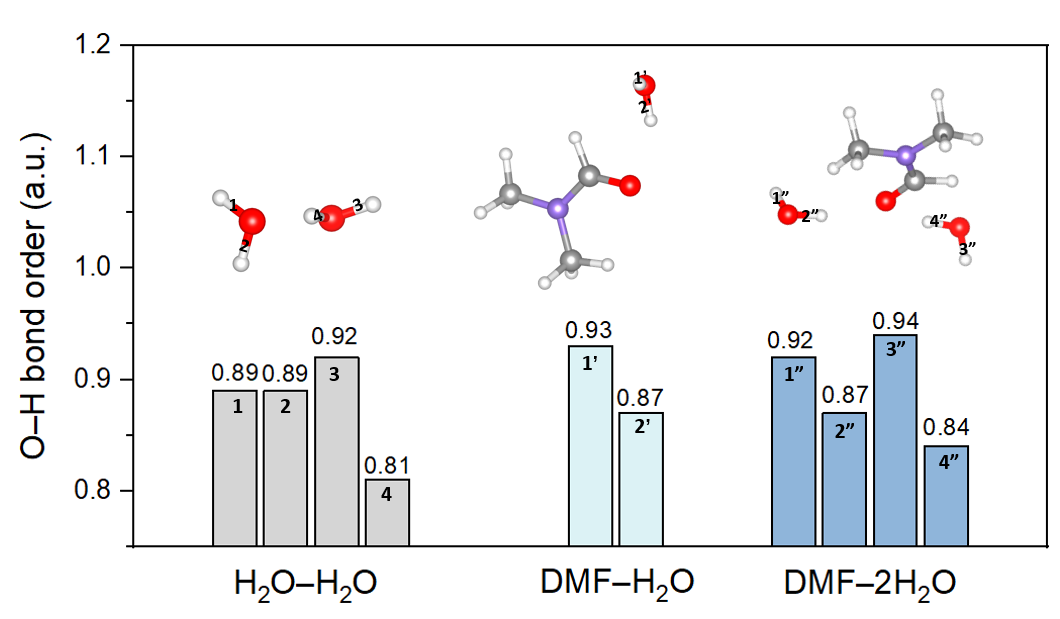


**Fig. S6.** The calculated O–H bond orders of H_2_O in H_2_O–H_2_O state, DMF–H_2_O state, and DMF–2H_2_O state.


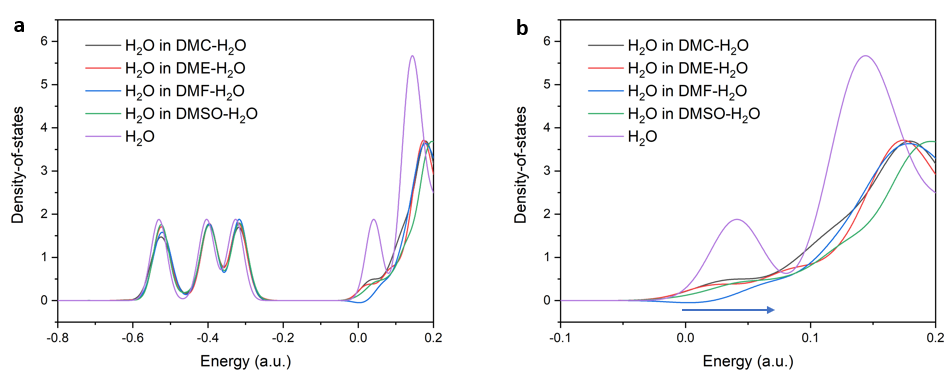


**Fig. S7.** The PDOS images (a) and magnified PDOS images (b) of H_2_O in DMC–H_2_O, DME–H_2_O, DMF–H_2_O, DMSO–H_2_O and H_2_O.


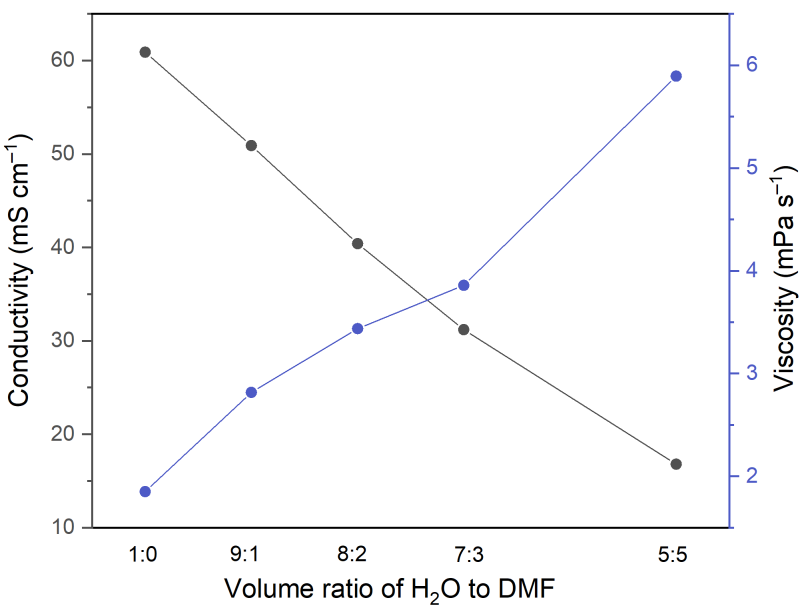


**Fig. S8.** Ionic conductivities and viscosities of different ZHD electrolyte.


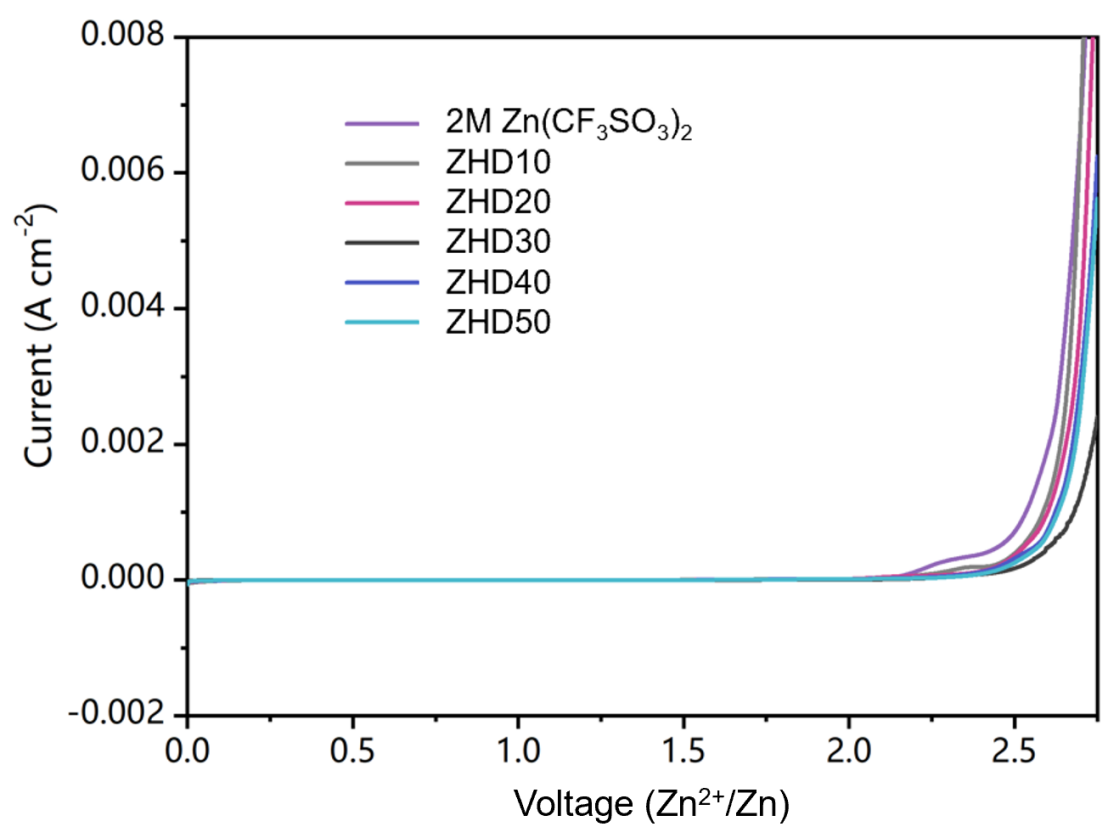


**Fig. S9.** LSV curves of asymmetric Zn||Ti cell in different ZHD electrolyte.


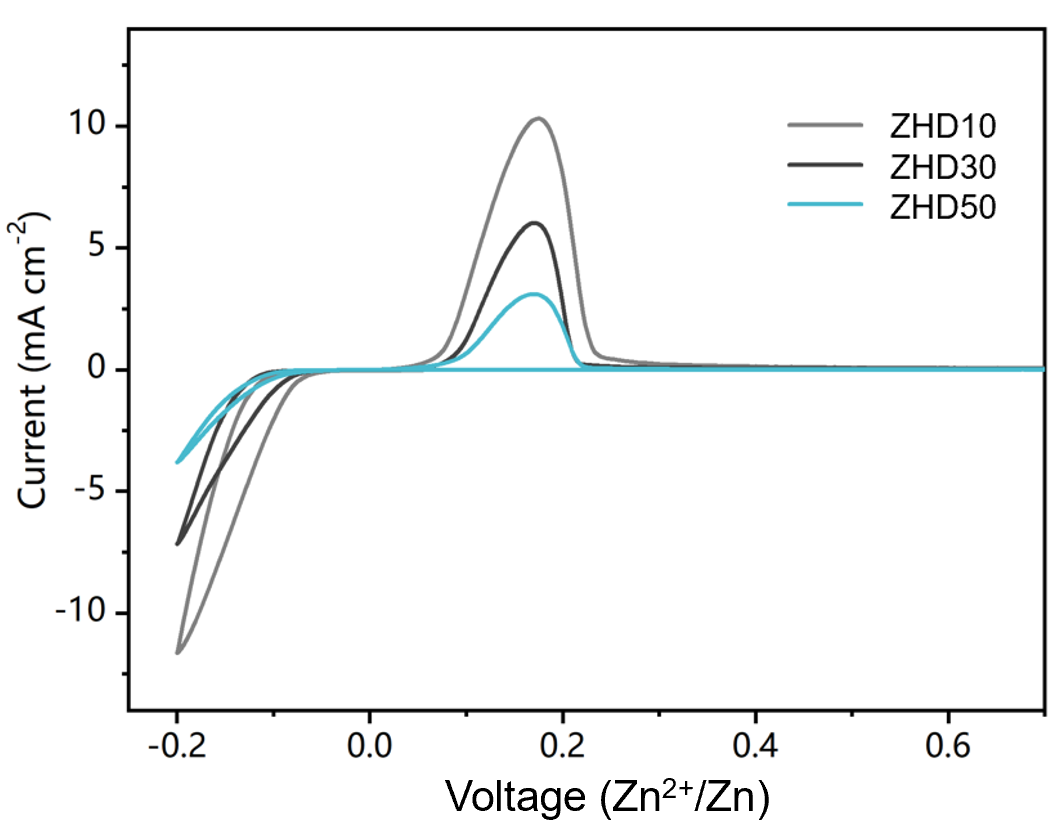


**Fig. S10.** CV curves of asymmetric Zn||Ti cell in different ZHD electrolyte.


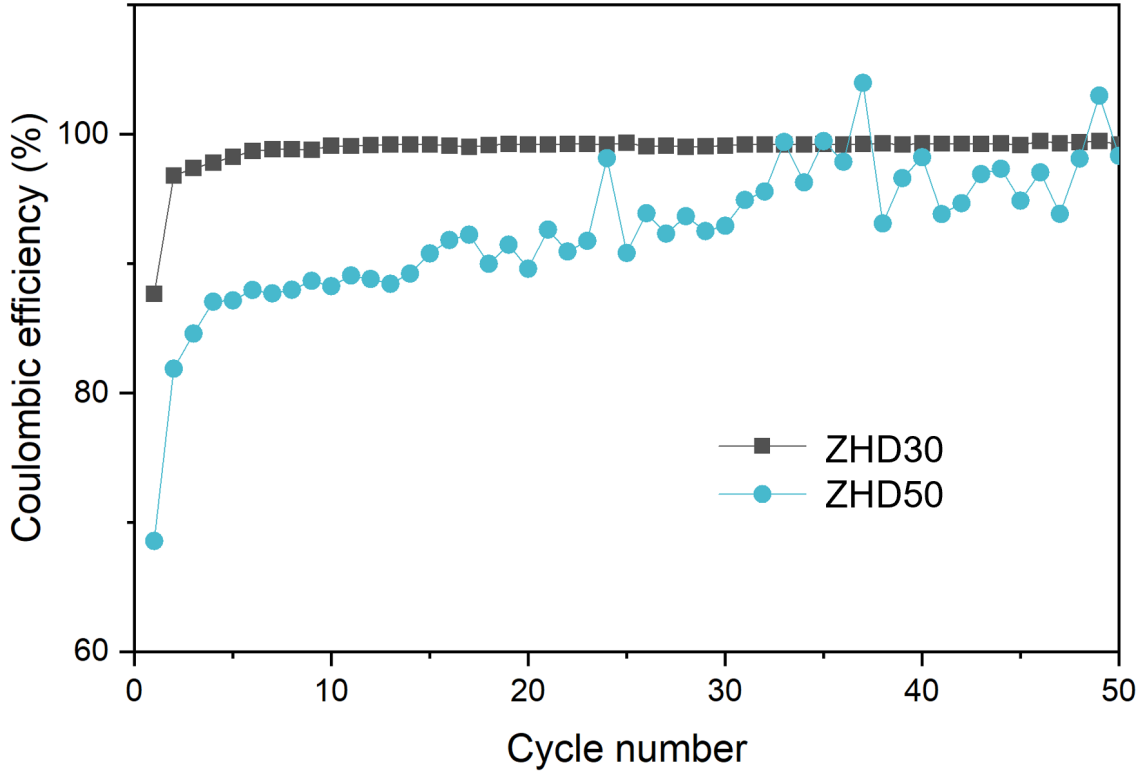


**Fig. S11.** CEs in Zn||Cu batteries with ZHD30 electrolyte and ZHD50 electrolyte.


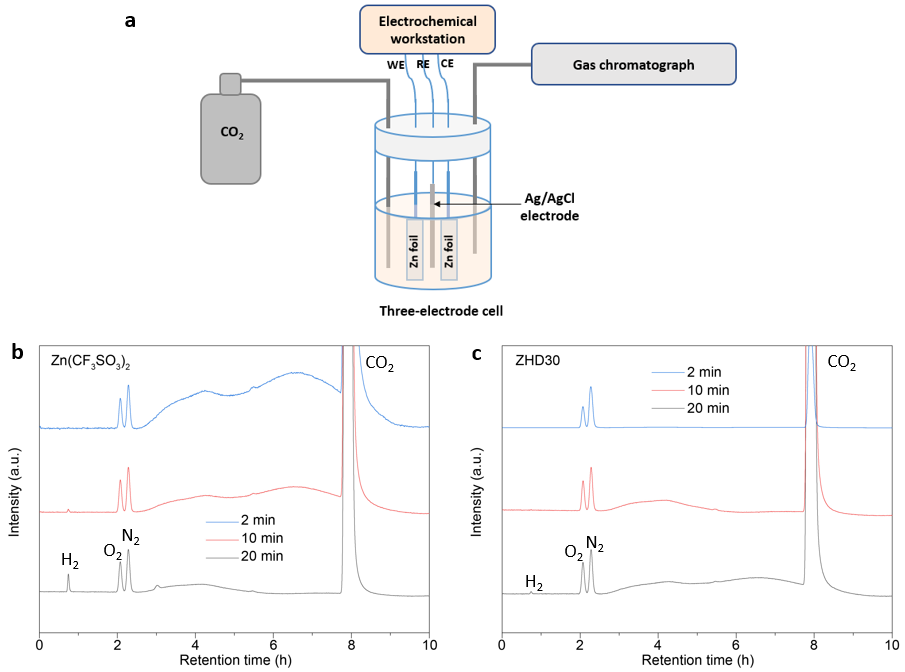


**Fig. S12.** The gas chromatography results of H-type glass cell separated by Nafion 117 membrane at 25 °C were conducted in 2 M Zn(CF_3_SO_3_)_2_ electrolyte and ZHD30 electrolyte during the Zn plating process. (a) The schematic of the combination of the gas chromatograph, three-electrode cell and the electrochemical workstation. (b) The results of gas chromatography in 2M Zn(CF_3_SO_3_)_2_ electrolyte. (c) The results of gas chromatography in ZHD30 electrolyte. The Zn foil (5 cm^–2^) was employed for the working and counter electrode. The reference electrode was the Ag/AgCl electrode. When Zn plated at a current density of 100 mA cm^–2^ for 2, 10, and 20 mins, the injection procedure of gas began.


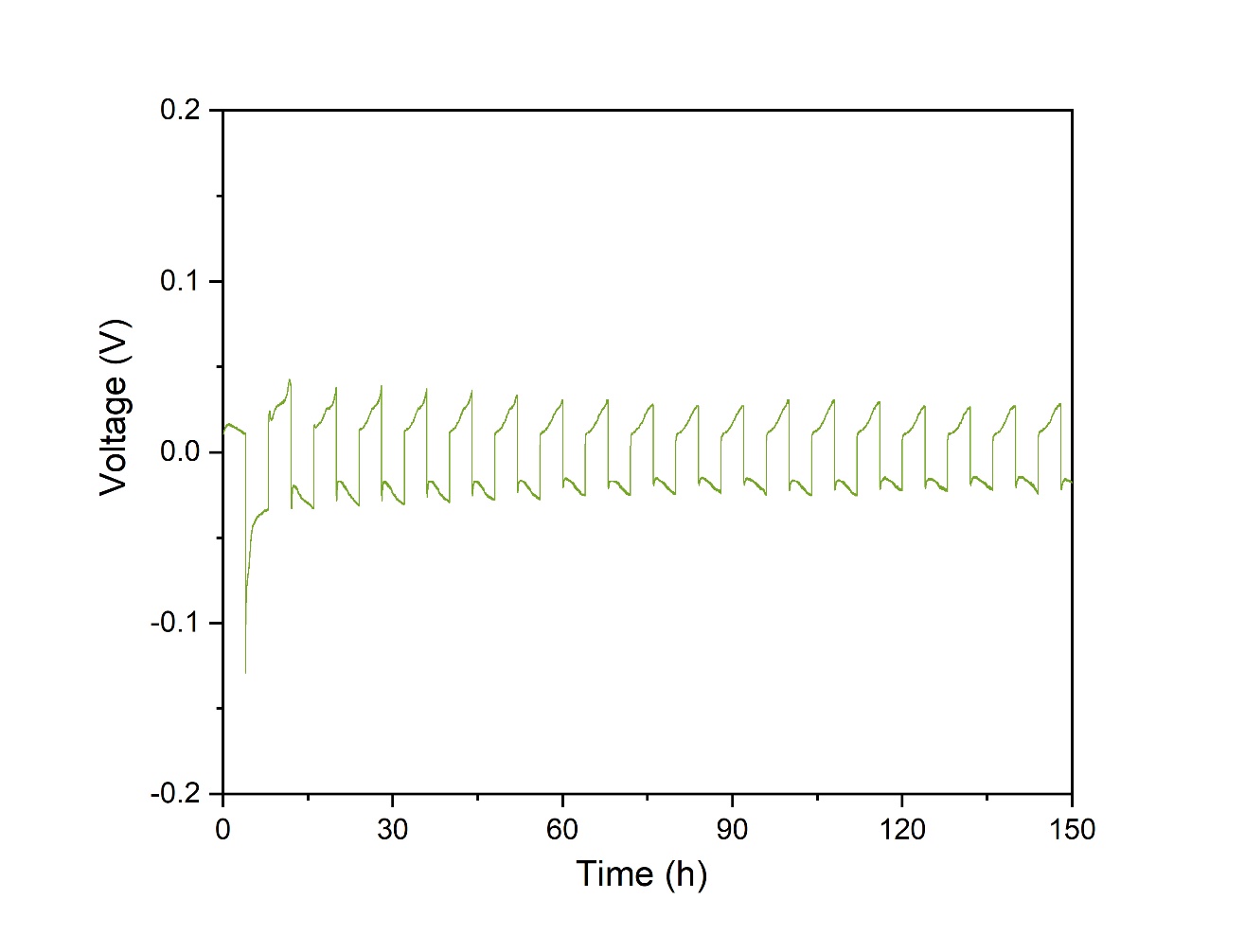


**Fig. S13.** Galvanostatic Zn plating/stripping curves in Zn||Zn symmetric cells at a current density of 0.5 mA cm^−2^ and an areal capacity of 2 mAh cm^−2^_._


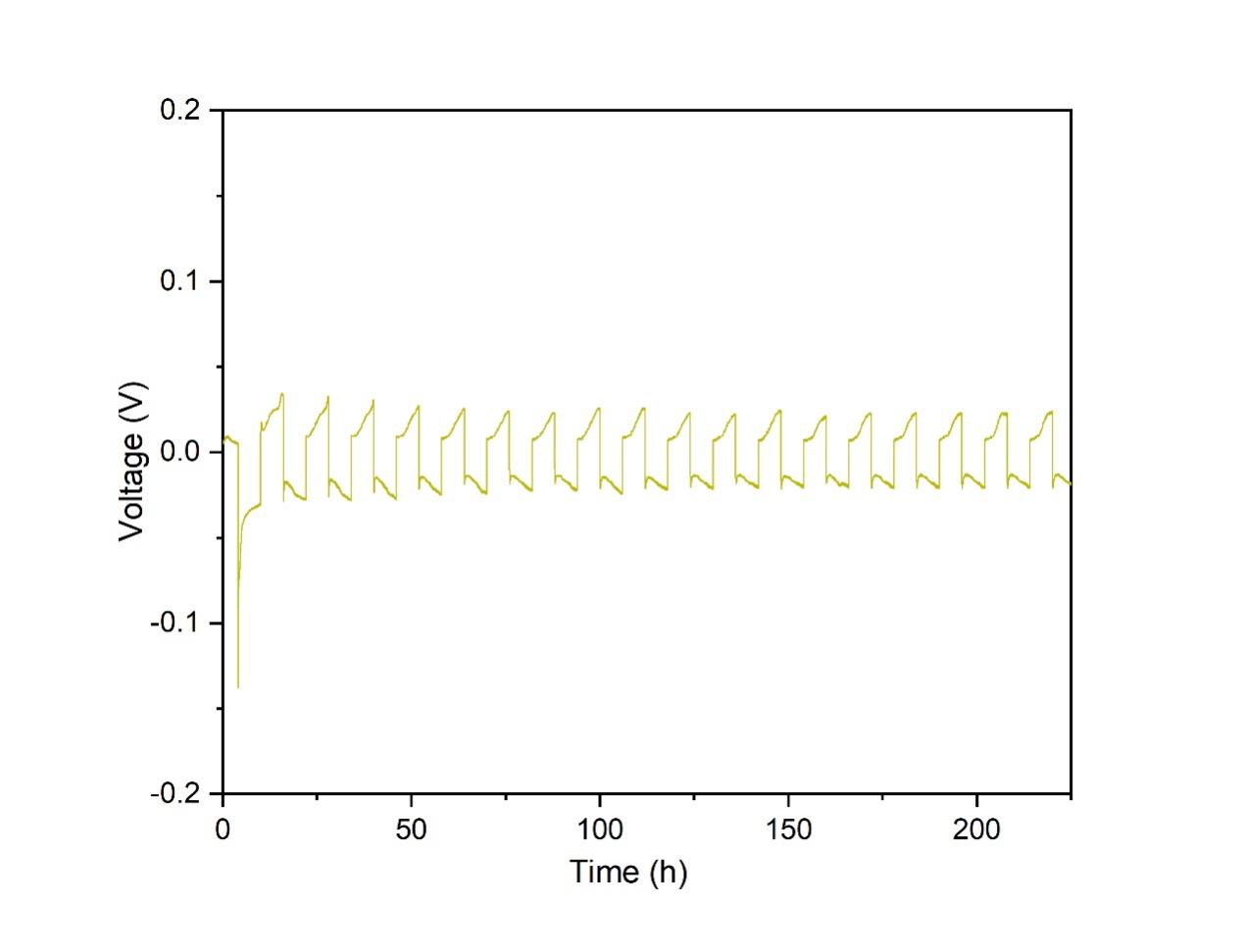


**Fig. S14.** Galvanostatic Zn plating/stripping curves in Zn||Zn symmetric cells at a current density of 0.5 mA cm^−2^ and an areal capacity of 3 mAh cm^−2^_._


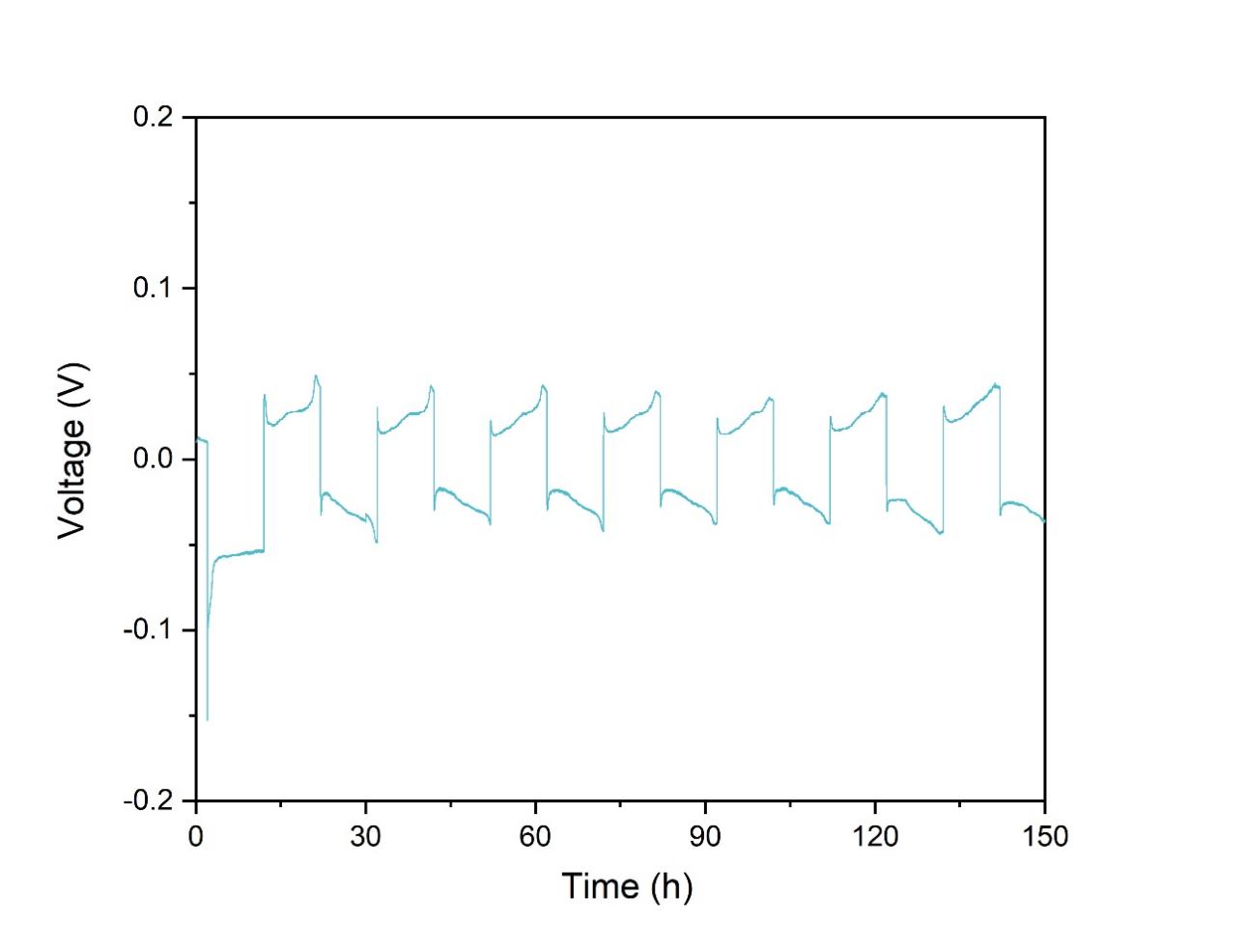


**Fig. S15.** Galvanostatic Zn plating/stripping curves in Zn||Zn symmetric cells at a current density of 0.5 mA cm^−2^ and an areal capacity of 5 mAh cm^−2^_._


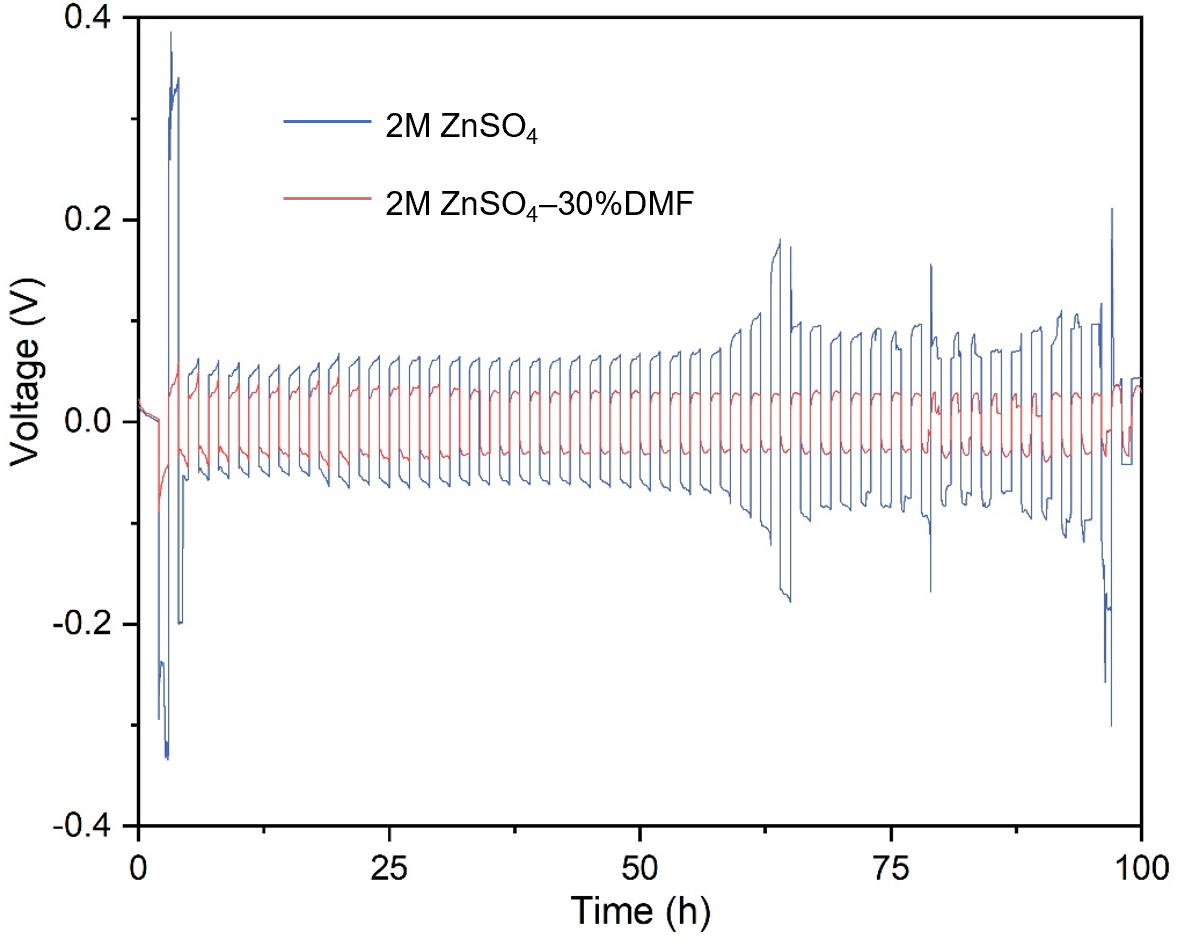


**Fig. S16.** Galvanostatic Zn plating/stripping curves in Zn||Zn symmetric cells at a current density of 0.5 mA cm^−2^ and a capacity of 0.5 mAh cm^−2^ in 2 M ZnSO_4_ electrolyte and 2 M ZnSO_4_–30%DMF electrolyte_._


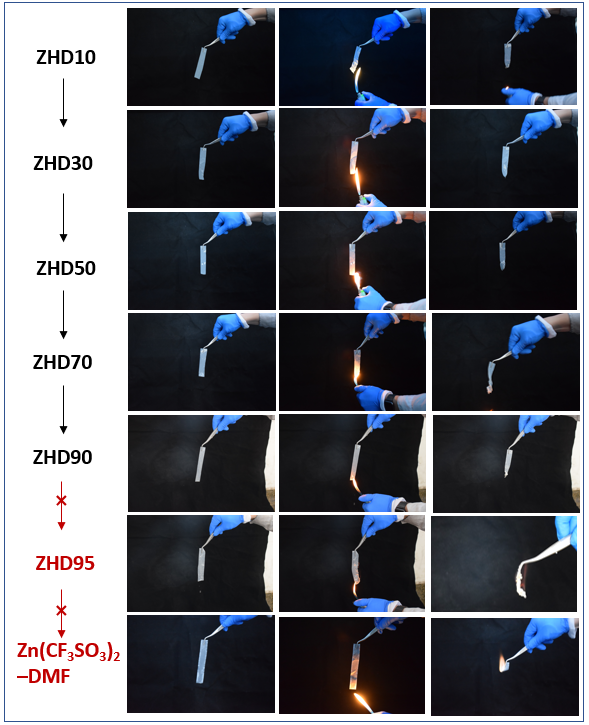


**Fig. S17.** Photographs of flammability tests of Celgard3501 saturated with ZHD10, ZHD30, ZHD50, ZHD70, ZHD90, ZHD95 electrolyte, and saturated Zn(CF_3_SO_3_)_2_ –DMF electrolyte.


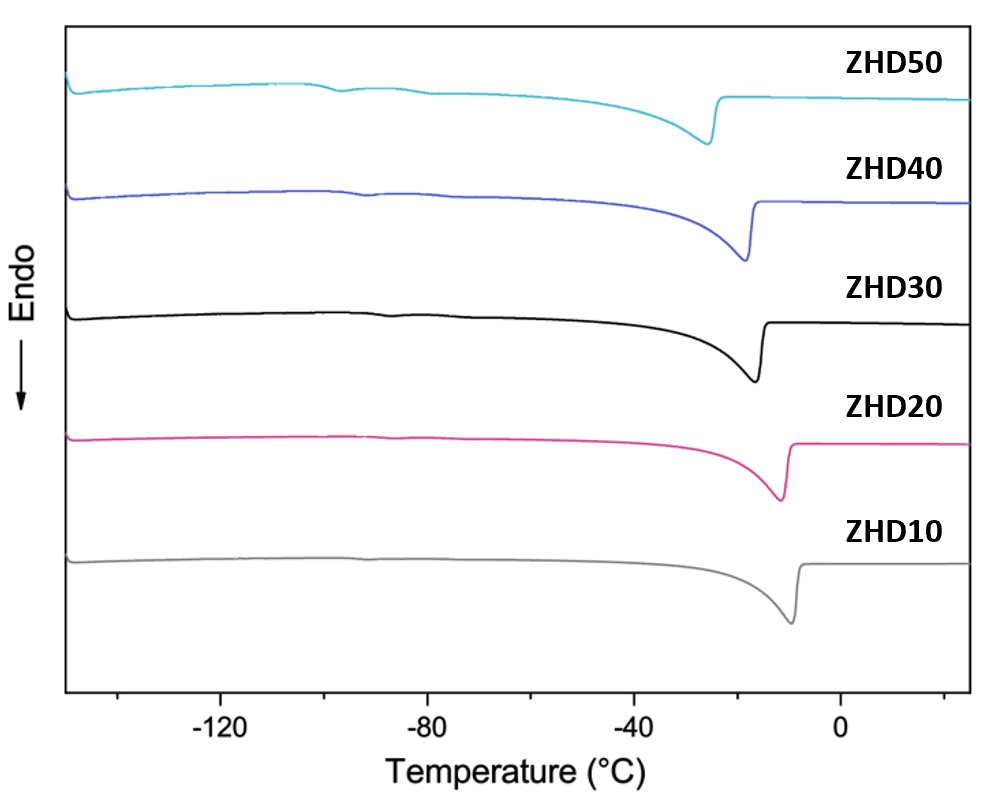


**Fig. S18.** DSC test from −150 to 25 °C at heating rate of 5 °C min^–1^ in different ZHD electrolyte.


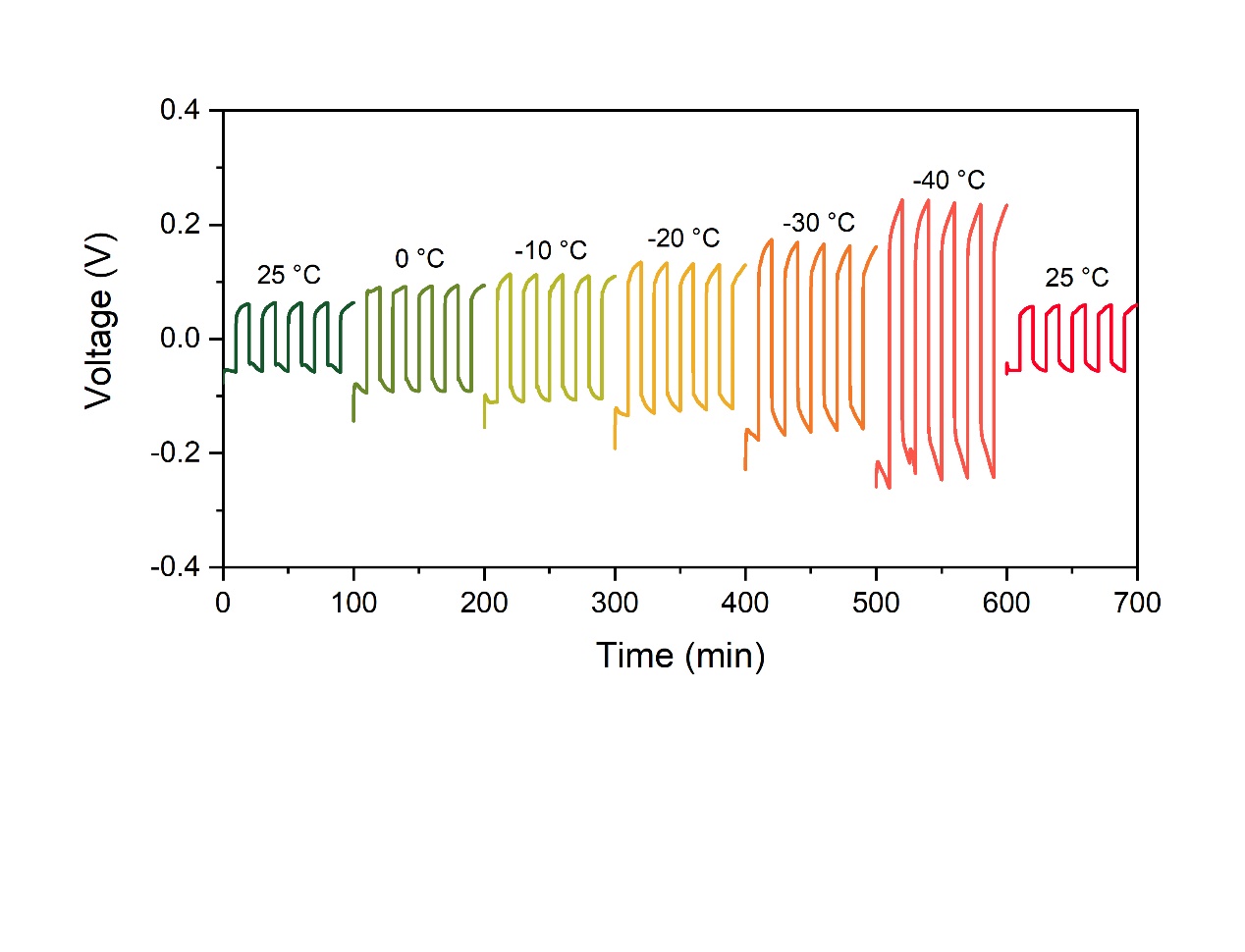


**Fig. S19.** Galvanostatic Zn plating/stripping curves in Zn||Zn symmetric cells based on ZHD30 electrolyte at 25, 0, –10, –20, –30 and –40 °C with the current density of 0.2 mA cm^–2^.


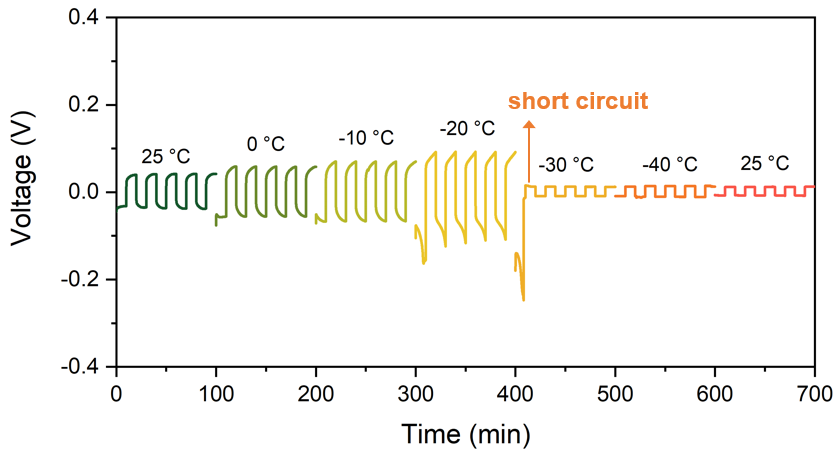


**Fig. S20.** Galvanostatic Zn plating/stripping curves in Zn||Zn symmetric cells based on 2 M Zn(CF_3_SO_3_)_2_ electrolyte at 25, 0, –10, –20, –30 and –40 °C with the current density of 0.2 mA cm^–2^.


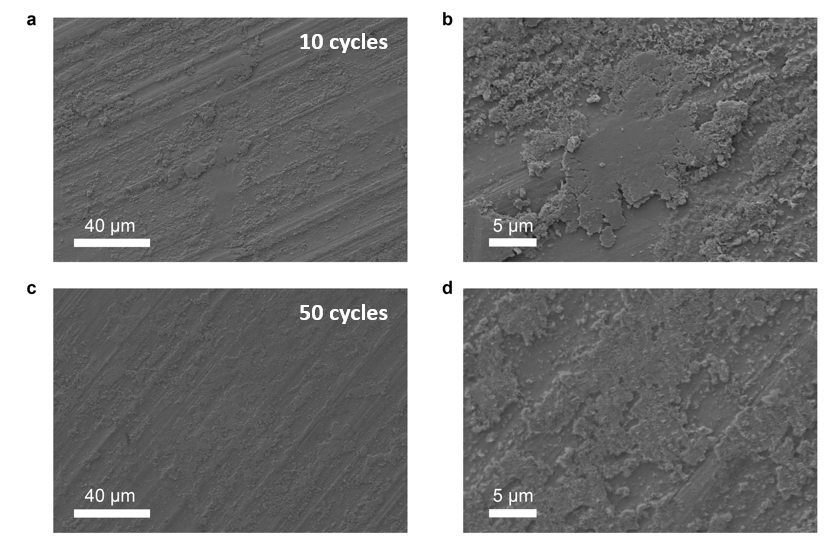


**Fig. S21.** SEM images (a) and magnified SEM images (b) of the deposited Zn metal in ZHD30 electrolyte after the 10^th^ plating at –40 °C. SEM images (c) and magnified SEM images (d) of the deposited Zn metal in ZHD30 electrolyte after the 50^th^ plating at –40 °C.


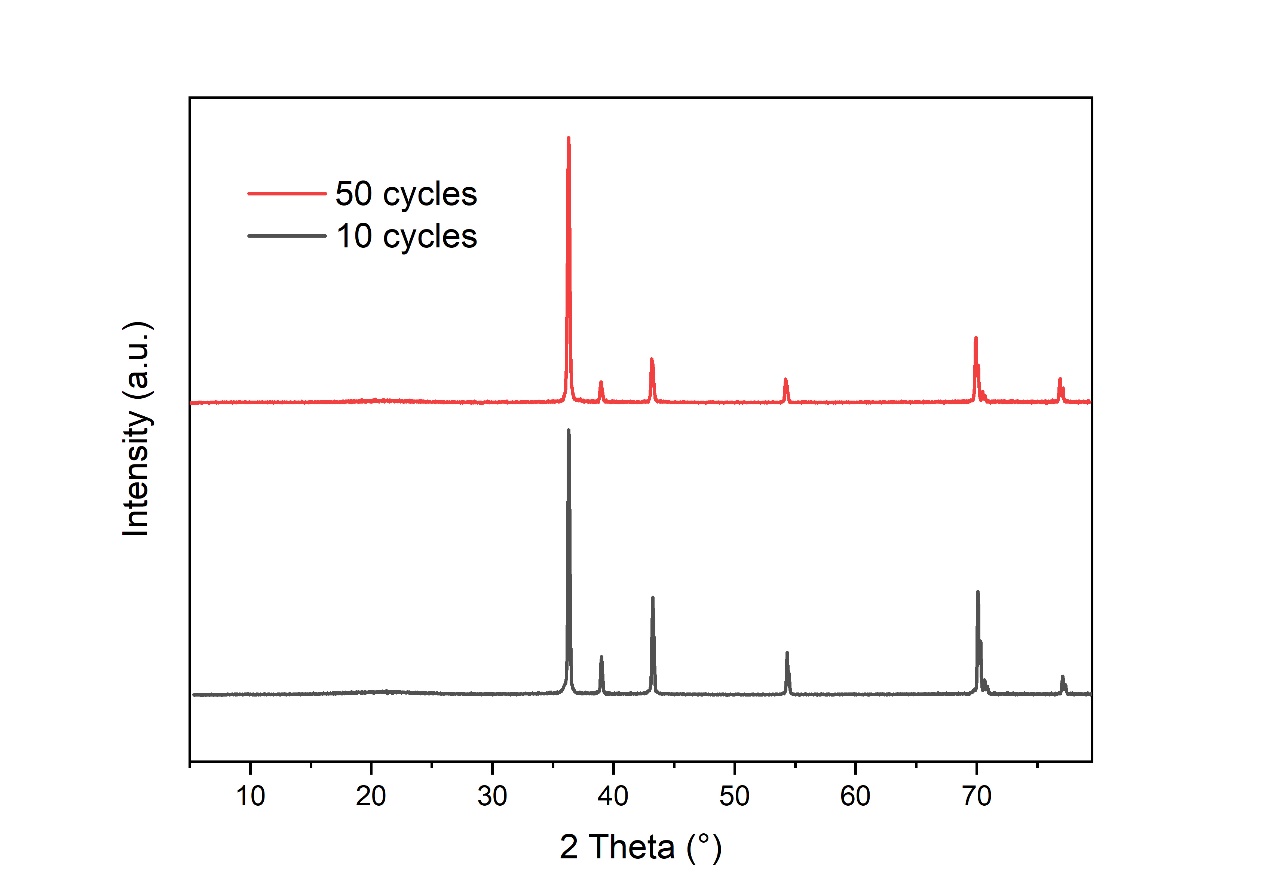


**Fig. S22.** XRD patterns of Zn anodes after plating/stripping cycles in ZHD30 electrolyte after the 10^th^ and 50^th^ plating at –40 °C.


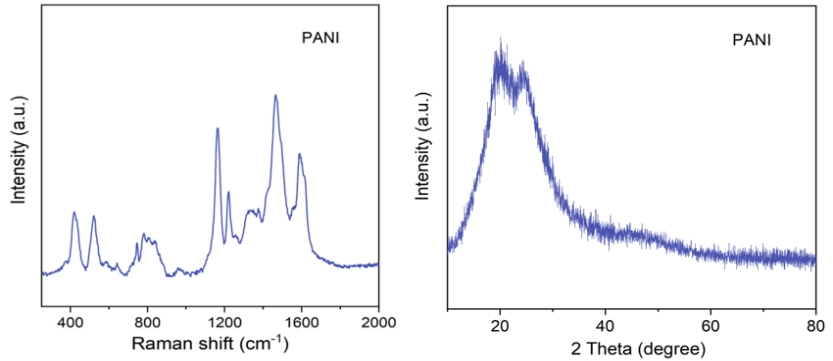


**Fig. S23.** Raman spectroscopy and XRD profile of as-prepared PANI.

In Raman spectrum, the stretching vibration peaks of C–C out-of-plane deformation at 420/518 cm^−1^, the stretching vibration peaks of out of-plane C–H motion at 806 cm^−1^, the C–H bending peak is at 1168 cm^−1^, the νC–N benzene diamine units at 1232 cm^−1^, the νC–N^+^ at 1334 cm^−1^, the νC=N at 1498 cm^−1^ and the C=N stretching vibration peak of benzenoid ring at 1591 cm^−1^. The diffraction peaks at 19.6° and 25.5° in XRD indicating the high purity of the synthetic PANI.


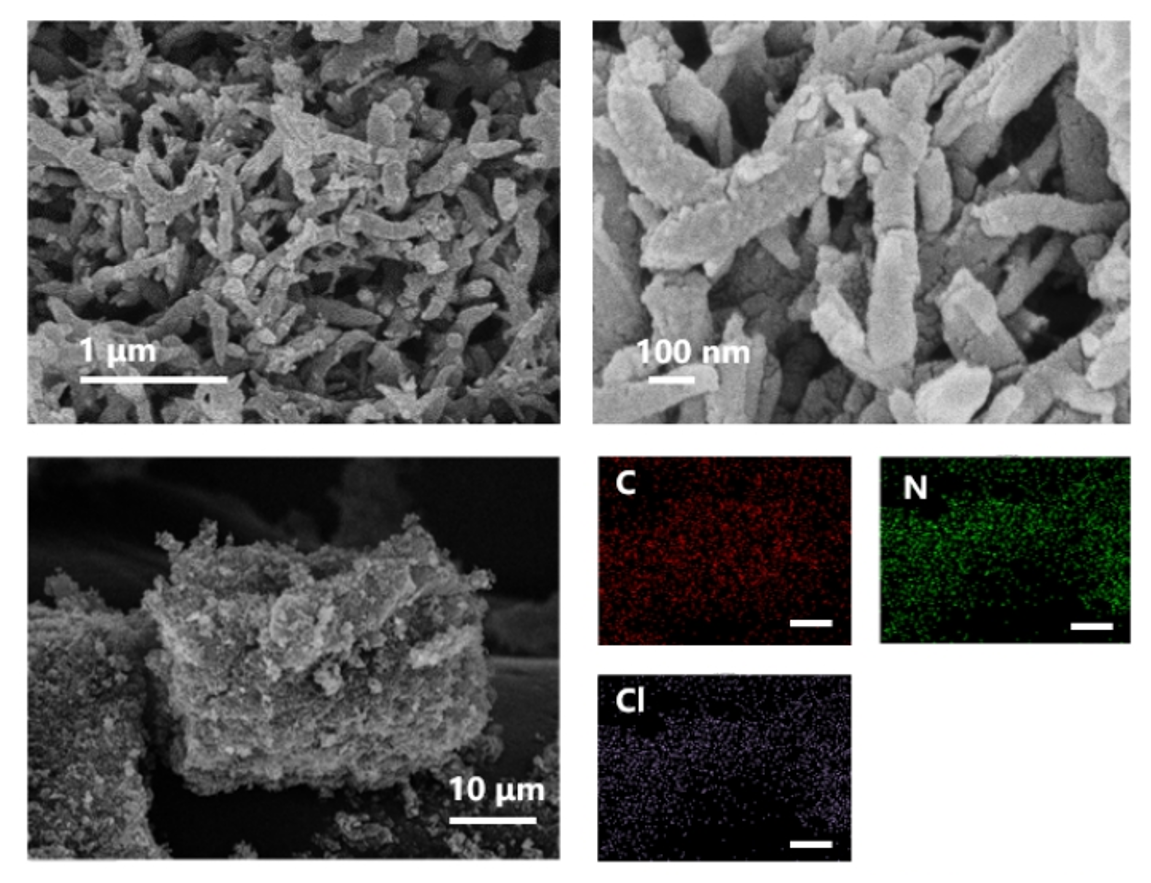


**Fig. S24.** SEM image, magnified SEM images and corresponding mapping of PANI.

**Table S1.** Parameters and compositions for molecular dynamic (MD) simulations of ZHD electrolytes.

|  | ZHD30 |
| --- | --- |
| Number of Zn^2+^ per box | 40 |
| Number of (CF_3_SO_3_)^–^ per box | 80 |
| Number of DMF per box | 80 |
| Number of H_2_O per box | 778 |
| Total number of atoms | 4752 |
| Simulation box size (Å^3^) | 3.453×3.453×3.453 |
| Bias temperature (K) | 298.15 |

**Table S2.** Summary of the atomic charge of every H atom in DMF–H_2_O (Figure S5).

|  | Average Atomic charge of H | Maximum Atomic charge of H |
| --- | --- | --- |
| H_2_O | 0.315674 | 0.315674 |
| H_2_O-H_2_O | 0.336936 | 0.397832 |
| DMF | 0.164502 | 0.170874 |
| DMF-H_2_O | 0.3199425 | 0.327931 |
| 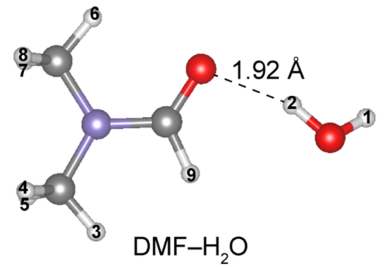 | **Atomic label** | **Atomic charge of H** |
|  | 1 | 0.311954 |
|  | 2 | 0.327931 |
|  | 3 | 0.166097 |
|  | 4 | 0.161753 |
|  | 5 | 0.166166 |
|  | 6 | 0.173611 |
|  | 7 | 0.166637 |
|  | 8 | 0.166619 |
|  | 9 | 0.156181 |

**Table S3.** Summary of the atomic charge of every H atom in DMF–2H_2_O (Figure S5).

|  | Average Atomic charge of H | | Maximum Atomic charge of H |
| --- | --- | --- | --- |
| H_2_O | 0.315674 | 0.315674 | |
| H_2_O-H_2_O | 0.336936 | 0.397832 | |
| DMF | 0.164502 | 0.170874 | |
| DMF-2H_2_O | 0.333489 | 0.38953 | |
| 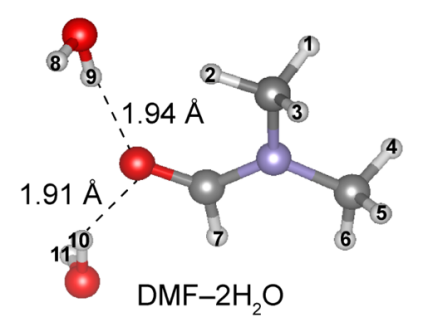 | **Atomic label** | **Atomic charge of H** | |
|  | 1 | 0.16993 | |
|  | 2 | 0.166597 | |
|  | 3 | 0.169459 | |
|  | 4 | 0.17408 | |
|  | 5 | 0.167111 | |
|  | 6 | 0.167043 | |
|  | 7 | 0.16465 | |
|  | 8 | 0.313589 | |
|  | 9 | 0.326328 | |
|  | 10 | 0.304509 | |
|  | 11 | 0.38953 | |

**Table S4.** Electronic conductivity and viscosity of different electrolyte (Figure S8).

| Electrolyte | Electronic conductivity  mS/cm | Viscosity  mPa s |
| --- | --- | --- |
| 2 M Zn(CF_3_SO_3_)_2_ | 60.9 | 1.85 |
| ZHD10 | 50.9 | 2.82 |
| ZHD20 | 40.4 | 3.44 |
| ZHD30 | 31.2 | 3.86 |
| ZHD50 | 16.8 | 5.89 |

**Table S5.** Summary of recently reported strategies for suppressing side reactions and boosting Zn reversibility in aqueous electrolyte.

| Electrolyte | Anode | Coulombic efficiency | Cycle life/Current density/Area capacity | Ref. |
| --- | --- | --- | --- | --- |
| 1M ZnSO_4_ | Zn foil coated with rGO | 95.0 | 200/1 mA cm^-2^/2 mAh cm^-2^ | 1 |
| 1 M ZnSO_4_ + 1000 ppm PEG | Zn foil | 99.6 | 570/1 mA cm^-2^/1 mAh cm^-2^ | 2 |
| 7.5 m ZnCl_2_ | Zn foil | 97.9 | 400/0.2 mA cm^-2^/0.2 mAh cm^-2^ | 3 |
| 30 m ZnCl_2_ | Zn foil | 95.4 | 600/0.2 mA cm^-2^/0.03 mAh cm^-2^ | 4 |
| 20 M LiTFSI + 1 M Zn(TFSI)_2_ | Zn powder | 99.5 | 170/0.2 mA cm^-2^/0.035 mAh cm^-2^ | 5 |
| 1 M ZnSO_4_+0.5 M Na_2_SO_4_ +1g L^-1^ PAM | Zn plated copper mesh | Nearly 100% | 350/0.2 mA cm^-2^/1 mAh cm^-2^ | 6 |
| Zn(ClO_4_)_2_·6H_2_O/SN ratio of 1:8 | Zn foil | 98.4 | 800/0.05 mA cm^-2^/0.5 mAh cm^-2^ | 7 |
| ZHD30 | **Zn foil** | **99.4** | **2000/0.5 mA cm^-2^/0.5 mAh cm^-2^** | **This work** |

**Supplemental References**

[1] C. Shen, X. Li, N. Li, K. Xie, J. Wang, X. Liu, B. Wei, ACS Appl. Mater. Interfaces 10 (2018) 25446-25453.

[2] X. Zhou, Y. Lu, Q. Zhang, L. Miao, K. Zhang, Z. Yan, F. Li, J. Chen, ACS Appl. Mater. Interfaces 12 (2020) 55476-55482.

[3] Q. Zhang, Y. Ma, Y. Lu, L. Li, F. Wan, K. Zhang, J. Chen, Nat. Commun. 11 (2020) 4463.

[4] C. Zhang, J. Holoubek, X. Wu, A. Daniyar, L. Zhu, C. Chen, D.P. Leonard, I.A. Rodríguez-Pérez, J.-X. Jiang, C. Fang, X. Ji, Chem. Commun. 54 (2018) 14097-14099.

[5] F. Wang, O. Borodin, T. Gao, X. Fan, W. Sun, F. Han, A. Faraone, J.A. Dura, K. Xu, C. Wang, Nat. Mater. 17 (2018) 543-549.

[6] Q. Zhang, J. Luan, L. Fu, S. Wu, Y. Tang, X. Ji, H. Wang, T, Angew. Chem. Int. Ed. 58 (2019) 15841-15847.

[7] W. Yang, X. Du, J. Zhao, Z. Chen, J. Li, J. Xie, Y. Zhang, Z. Cui, Q. Kong, Z. Zhao, C. Wang, Q. Zhang, G. Cui, Joule, 4 (2020) 1557-1574.
